# Attributable fraction of tobacco smoking on cancer using population-based nationwide cancer incidence and mortality data in Korea

**DOI:** 10.1186/1471-2407-14-406

**Published:** 2014-06-06

**Authors:** Sohee Park, Sun Ha Jee, Hai-Rim Shin, Eun Hye Park, Aesun Shin, Kyu-Won Jung, Seung-Sik Hwang, Eun Shil Cha, Young Ho Yun, Sue Kyung Park, Mathieu Boniol, Paolo Boffetta

**Affiliations:** 1 Division of Cancer Registration and Surveillance, National Cancer Center, Goyang, Korea; 2 Department of Biostatistics, Graduate School of Public Health, Yonsei University, Seoul, Korea; 3 Department of Epidemiology and Health Promotion, Institute for Health Promotion, Graduate School of Public Health, Yonsei University, Seoul, Korea; 4 Western Pacific Regional Office, World Health Organization, Manila, Philippines; 5 Department of Preventive Medicine, Seoul National University College of Medicine, Seoul, Korea; 6 Department of Social and Preventive Medicine, School of Medicine, Inha University, Incheon, Korea; 7 Department of Preventive Medicine, College of Medicine, Korea University, Seoul, Korea; 8 College of Medicine, Seoul National University, Seoul, Korea; 9 Department of Preventive Medicine, College of Medicine, Seoul National University, Seoul, Korea; 10 International Prevention Research Institute, Lyon, France; 11 The Tisch Cancer Institute, Mount Sinai School of Medicine, New York, NY, USA

**Keywords:** Risk factor, Population attributable fraction, Lifestyle, Asia

## Abstract

**Background:**

Smoking is by far the most important cause of cancer that can be modified at the individual level. Cancer incidence and mortality rates in Korea are the highest among all Asian countries, and smoking prevalence in Korean men is one of the highest in developed countries. The purpose of the current study was to perform a systematic review and provide an evidence-based assessment of the burden of tobacco smoking-related cancers in the Korean population.

**Methods:**

Sex- and cancer-specific population-attributable fractions (PAF) were estimated using the prevalence of ever-smoking and second-hand smoking in 1989 among Korean adults, respectively, and the relative risks were estimated from the meta-analysis of studies performed in the Korean population for ever-smoking and in the Asian population for passive smoking. National cancer incidence data from the Korea Central Cancer Registry and national cancer mortality data from Statistics Korea for the year 2009 were used to estimate the cancer cases and deaths attributable to tobacco smoking.

**Results:**

Tobacco smoking was responsible for 20,239 (20.9%) cancer incident cases and 14,377 (32.9%) cancer deaths among adult men and 1,930 (2.1%) cancer incident cases and 1,351 (5.2%) cancer deaths among adult women in 2009 in Korea. In men, 71% of lung cancer deaths, 55%–72% of upper aerodigestive tract (oral cavity, pharynx, esophagus and larynx) cancer deaths, 23% of liver, 32% of stomach, 27% of pancreas, 7% of kidney and 45% of bladder cancer deaths were attributable to tobacco smoking. In women the proportion of ever-smoking-attributable lung cancer was 8.1%, while that attributable to second-hand smoking among non-smoking women was 20.5%.

**Conclusions:**

Approximately one in three cancer deaths would be potentially preventable through appropriate control of tobacco smoking in Korean men at the population level and individual level. For Korean women, more lung cancer cases and deaths were attributable to second-hand than ever-smoking. Effective control programs against tobacco smoking should be further developed and implemented in Korea to reduce the smoking-related cancer burden.

## Background

Smoking is by far the most important single cause of cancer in high-income countries [1]. According to the International Agency for Research on Cancer (IARC), tobacco smoking causes cancers of the oral cavity, pharynx, esophagus, stomach, colon, rectum, liver, pancreas, larynx, lung, cervix, kidney, bladder, ureter, and bone marrow [2]. The first attempt to estimate the global burden of cancer was performed by Doll and Peto using US data, which provided the population-attributable fraction (PAF) of smoking for cancer mortality [3]. Since then, only a few studies have attempted to estimate the relative importance of cancer risk factors including the updated estimates of year 2000 [4-6]. Most previous estimates of attributable cancers have been conducted in high-resource countries, primarily Western countries and only a few studies were conducted in Asian countries [7,8].

Smoking patterns and the magnitude of the increased risk of lung cancer among smokers are very different in Asian populations compared with those in Western populations. The relative risks (RRs) of lung cancer observed among Asian smokers are generally lower than those in the Western population. Several possible explanations for the differences in RRs among Asians and individuals in Western countries have been suggested. They are a longer duration of heavy smoking in Americans, a more toxic formulation of American-manufactured cigarettes, a higher efficiency of filters in Japanese cigarettes, lower alcohol consumption by Japanese males, differences in genetic susceptibility to tobacco carcinogens, and a higher background risk of lung cancer among non-smokers [9]. The lung cancer mortality rates among non-smokers in the Asian population (rate = 35.6 in Japanese men and 24.6 in Japanese women) were indeed shown to be higher than those in the US (rate =15.7 in a CPS-I study and 14.7 in a CPS-II study) [10,11]. This raises an important question regarding whether it would be appropriate to apply the PAF estimated from studies performed in Western populations to other countries. Thus, it would seem to be essential to develop an estimate of the PAF of risk factors for cancer that are specific to each region of the world.

Cancer is the leading cause of death in Korea; one in every four Koreans becomes a victim of this life-threatening disease. Furthermore, Korea has the highest cancer incidence and mortality rates among all East Asian countries [12]. Among the evaluated cancer risk factors, smoking is known to be the most important factor that can be modified at the individual level. Smoking prevalence has been very high among Korean men. Although having continuously decreased from 70.8% in 1992 to 46.7% in 2009 (Additional file 1: Figure S1), the smoking prevalence in Korean men is still among the highest in member countries of the Organisation for Economic Co-operation and Development (OECD) [13,14]. Given very different patterns in the relative risks of tobacco smoking for cancer in Asian and Western countries, the importance of evaluating the region-specific PAF for smoking in cancer should be recognized to develop cancer prevention strategies tailored to each country. The objective of the present study, thus, was to perform a systematic review and provide an evidence-based assessment of cancer incident cases and deaths attributable to tobacco smoking, including both ever-and second-hand smoking, in the Korean population using nationwide cancer incidence and mortality data.

## Methods

### Definition of exposure

Tobacco smoking status was classified as “never”, “former”, and “current” in this study. To describe the cancer burden due to tobacco smoking, we considered “ever-smoking” and “second-hand smoking”. We used the term “ever-smoking” to mean “former” or “current” smoking. Duration of smoking and cumulative consumption (“pack-years”) were not considered in the overall calculation of PAF. Exposure to second-hand smoking was considered as exposed to smoking in the household (smoking spouse or other family members) and/or at the workplace. “Smokeless tobacco” is hardly consumed in Korea, and thus was not taken into consideration in this study. Because smoking is a risk factor that can be avoided or completely suppressed, at least in theory, PAF was estimated under the alternative scenario of total absence of exposure [15].

### Smoking prevalence in Korea

The burden of cancer observed in 2009 reflects past exposure to risk factors. We assumed a latency period of approximately 20 years between smoking exposure and cancer occurrence. We estimated the adult smoking prevalence separately by gender using the Korea National Health Examination Survey (KNHES) performed in 1989. The KNHES is a national survey on a random sample of Koreans, designed to provide reliable nationwide statistics on the state of health, health-related behavior, and perceptions. The prevalence rates of 11.7% of former smokers and 70.8% of current smokers in Korean men and 0.3% of former smokers and 3.9% of current smokers in Korean women in 1989 were used [16]. A representative survey on second-hand smoking has only been available from the Korea National Health and Nutrition Examination Survey (KNHANES) in recent years (Additional file 1: Figure S2). We used the data for second-hand smoking prevalence from 2007 to 2012 from KNHANES, and the prevalence of current smoking during 2007–2012 to extrapolate the passive smoking prevalence in 1989 through fitting a log-linear regression model [17]. Because KNHES and KNHANES data do not contain personal information and are publically available through on-line request (http://knhanes.cdc.go.kr/knhanes/), we did not have to address ethical concerns.

### Relative risk of tobacco smoking

Relative risks (RRs) of smoking-related cancers were evaluated for current smokers and former smokers compared with never smokers from the analysis of a large-scale population-based prospective study or by performing a meta-analysis. The studies reporting RRs of smoking and cancer published before August 1, 2012 were identified using databases including PubMed (http://www.ncbi.nlm.nih.gov/pubmed/) and KoreaMed (http://www.koreamed.org/SearchBasic.php). The search keywords were “Korea”, “Asia”, “smoking”, “tobacco smoking”, “passive smoking”, “secondhand smoke”, “environmental tobacco smoke”, “risk”, and “cancer”. Language was limited to English or Korean. At least two independent investigators performed literature search and reviewed articles. Additional citations were identified from the references of searched articles and information given by cancer experts in Korea. When there were multiple reports of a same study, publication with the longest follow-up period or the largest event numbers was selected for estimation of pooled RRs, to avoid bias. For ever-smoking, 105 studies were initially identified, but many of them were excluded in the final analysis for several reasons: risk estimates with precision information (e.g., standard error, 95% confidence interval [CI]) were not available, the classification for smoking was different than never, former, and current smokers (53 studies), and multiple results were reported from the same study population (13 studies). Nineteen studies were used in the final analysis to estimate the pooled RRs for ever-smoking and the RRs for most cancer sites were estimated from a few studies including a large-scale cohort study [18-36]. When possible, the pooled RRs were separately estimated for cancer incidence and mortality. If a separate RR estimate for cancer mortality was not available, we used the RR for cancer incidence in place of RR for cancer mortality based on the assumption that tobacco smoking does not affect cancer survival. When the estimated RR was lower than one, we replaced the RR by one because the cancer sites we considered in this study are the ones that were convincingly classified as carcinogens to human. Additional analysis results on RRs from updated datasets with a longer follow-up period and analyses adjusting for confounding variables such as age and alcohol drinking were obtained through personal communication with the authors of cited publications [21,27,31,35].

For oral cavity, pharynx, stomach and colorectal cancer, there was no reliable RR estimates for women, hence RR of men was used for women instead. For estimation of the RR for ever-smoking we used only studies conducted in the Korean population (Additional file 1: Table S1 and S2). However, for second-hand smoking, as the number of Korean studies was limited, the study results from other Asian countries such as China and Japan were considered in order to obtain reliable estimates. In total, risk estimates from 19 studies were used for the meta-analysis where pooled RR estimate of second-hand smoking were calculated (Additional file 1: Table S3 and S4, Additional file 1: Figure S3–S6) [37-58].

Meta-analyses were performed to estimate the pooled RRs and 95% confidence intervals (CIs) based on both fixed- and random-effects models. To check for heterogeneity, Q statistics and Higgin’s I^2^ value was used. We considered that there existed heterogeneity among studies if the Q statistics was significant (p < 0.05) or I^2^ value was above 75%. In case of heterogeneity, the risk estimates from a random-effects model were used. Publication bias was checked by funnel plot and Begg’s test. The “Metan” command in Stata (ver. 10.0; StataCorp, College Station, TX, USA) and Comprehensive Meta-Analysis version 2 (Biostat, Englewood, NJ, USA) were used to perform the meta-analysis.

### Cancer incidence and mortality data

The number of cancer incidence cases in 2009 in Korea was obtained from the Korea Central Cancer Registry, a population-based nationwide cancer registry in Korea [59]. Similarly, the number of cancer deaths in 2009 was obtained using death certificate data from Statistics Korea [60]. The cancers of interest were those that showed convincing evidence for a positive association with tobacco smoking and for which relative risk estimates in Korea were available. Such cancers included oral cavity, pharynx, esophagus, stomach, colorectum, liver, pancreas, larynx, lung, cervix uterine, ovary, kidney, and bladder [61]. For second-hand smoking, the only cancer retained in the analysis was lung cancer among never-smokers. When applying PAF to cancer incidence cases and deaths, we only used the number of cases and deaths aged 20 years and older because when assuming a latency of 20 years, tobacco causes no cancers below age 20 years, and the RRs and smoking prevalence data reported in the literature were estimated from adult study populations. Because we used the aggregated data that do not contain personal information and that are publically available through website (http://www.cancer.go.kr for cancer incidence statistics; and http://www.kosis.kr for cancer mortality statistics), we did not have to address ethical concerns.

### Estimation of population attributable fraction

Estimation of attributable causes of cancer was made through the proportion of cancers in the total population that was attributable to a specific risk factor. The PAF was calculated by the following Levin’s formula for multiple categories (k), as proposed by Hanley [62,63]:

$$\mathrm{PAF}\;\left(\%\right)=\frac{\sum_{k=1}^{K}p_{k}\;\left({\mathrm{RR}}_{k}-1\right)}{\sum_{k=1}^{K}p_{k}\;\left({\mathrm{RR}}_{k}-1\right)+1}\times 100,k=1,2,\ldots ,K$$

where *RR* is the relative risk of cancer for smoking, *p* is the smoking prevalence in the total adult population (aged 20+ years), and *K* is the number of categories in the smoking exposure.

The joint effect of second-hand smoking in the household and workplace was taken into account by assuming the independence of exposure from two sources as follows:

$$\begin{array}{ll} \mathrm{PA}F_{\mathrm{HW}}= & \mathrm{PA}F_{H}\times \mathrm{PA}F_{W}+\mathrm{PA}F_{H}\left(1-\mathrm{PA}F_{W}\right)\\ & +\mathrm{PA}F_{W}\left(1-\mathrm{PA}F_{H}\right) \end{array}$$

where *PAF*_*H*_ and *PAF*_*W*_ are the PAFs for passive smoking exposure in the household and workplace, respectively [64]. Derivation of second-hand smoking-related lung cancer cases and deaths among never smokers is demonstrated in Additional file 1: Table S5.

### Sensitivity analysis for the estimation of population attributable fraction (PAF) of tobacco smoking

To account for the uncertainty in PAF estimation arising from the estimation of RRs for each cancer site, a sensitivity analysis was performed under alternative scenarios using the lower and upper limits of the 95% CIs of RR estimates.

## Results

Among all cancer sites reviewed in this study, laryngeal cancer had the highest RR estimate (RR = 4.65 for current smoking men and 9.10 for current smoking women for cancer incidence; RR = 4.50 for current smoking men and RR = 3.60 for current smoking women for cancer mortality, Table 1). The RR for lung cancer mortality among current smokers was estimated to be 4.40 for men and 3.20 for women. For other cancer sites, the RRs for current smokers ranged from 1.10 to 6.70 for cancer incidence and from 1.10 to 3.30 for cancer mortality, except for a few cases where the RR was estimated to be less than one with insignificant p-values (Table 1). The results from meta-analysis showed that the effect of second-hand smoking on lung cancer incidence for men was not significant, however, that for women showed a significantly elevated risk of lung cancer incidence (RR = 1.32 for second-hand smoking at home; RR = 1.37 for second-hand smoking at workplace, Table 2). Second-hand smoking at home or in the workplace was responsible for 20.7% of lung cancer incidence and 20.5% of lung cancer mortality among never-smoking women (994 lung cancer cases and 726 deaths). Among never-smoking men, 66 lung cancer cases and 57 deaths were attributable to passive smoking which showed a much lower PAF (5.9% for lung cancer incidence and 10.5% for lung cancer mortality) in men than that in women (Table 2).

**Table 1:** Relative risk for tobacco smoking and cancer in Korea

**Cancer site (ICD-10)**	**Gender**	**Pooled RR (95% CI)**				**Sources of pooled RR (OR)**
		**Incidence**		**Mortality**		
		**Former smokers**	**Current smokers**	**Former smokers**	**Current smokers**	
Oral Cavity (C00-C09)^a^	Men	1.03 (0.63-1.68)	2.19 (1.54-3.12)	0.80 (0.10-13.50)	3.30 (0.50-34.60)	[20,21]
	Women	-	6.70 (1.10-39.40)^b^	0.80 (0.10-13.50)^c^	3.30 (0.50-34.60)^c^	[21]
Pharynx (C10-C14)^a^	Men	1.03 (0.63-1.68)	2.19 (1.54-3.12)	0.80 (0.10-13.50)	3.30 (0.50-34.60)	[20,21]
	Women	-	6.70 (1.10-39.40)^b^	0.80 (0.10-13.50)^c^	3.30 (0.50-34.60)^c^	[21]
Esophagus (C15)	Men	1.20 (1.05-1.37)	2.23 (1.99-2.50)	1.45 (1.21-1.73)	2.64 (2.25-3.09)	[20,21,26]
	Women	1.10 (0.40-3.10)	1.60 (0.80-3.10)	1.60 (0.60-5.10)	0.90 (0.30-2.70)	[21]
Stomach (C16)	Men	1.22 (0.87-1.71)	1.51 (1.46-1.55)	1.31 (1.21-1.41)	1.60 (1.51-1.71)	[20,21,23,27,28,34]
	Women	1.22 (0.87-1.71)^c^	1.51 (1.46-1.55)^c^	1.01 (0.83-1.24)	1.04 (0.85-1.26)	[21,27]
Colorectum (C18-C20)	Men	1.13 (1.02-1.26)	0.98 (0.78-1.23)	1.10 (0.90-1.40)	1.10 (0.80-1.40)	[20,21,24,33]
	Women	1.07 (0.70-1.63)	0.97 (0.76-1.25)	1.10 (0.90-1.40)^c^	1.10 (0.80-1.40)^c^	[24,33]
Liver (C22)	Men	1.20 (1.10-1.30)	1.40 (1.30-1.50)	1.20 (1.00-1.30)	1.40 (1.30-1.60)	[21]
	Women	0.80 (0.10-5.60)	2.50 (1.00-6.30)	1.90 (0.30-14.20)	2.60 (0.60-11.00)	[21]
Pancreas (C25)	Men	1.20 (1.00-1.40)	1.50 (1.30-1.70)	1.11 (0.93-1.33)	1.50 (1.31-1.71)	[21,27]
	Women	0.80 (0.50-1.10)	1.20 (0.90-1.50)	0.90 (0.5-1.20)	1.10 (0.80-1.40)	[21]
Larynx (C32)	Men	2.01 (1.49-2.73)	4.65 (3.61-6.00)	1.70 (1.00-2.90)	4.50 (2.80-7.10)	[20,21]
	Women	0.90 (0.10-6.80)	9.10 (4.60-17.80)	0.90 (0.10-6.90)	3.60 (1.30-9.70)	[21]
Lung (C33-C34)	Men	1.21 (0.87-1.69)	2.58 (1.83-3.63)	1.82 (1.63-2.03)	4.40 (3.98-4.87)	[19-21,25,27,30,31,36]
	Women	1.65 (1.37-1.99)	2.37 (2.09-2.68)	1.90 (1.50-2.40)	3.20 (2.70-3.70)	[21,31]
Cervix uteri (C53)	Women	1.15 (0.87-1.52)	1.12 (0.92-1.35)	1.20 (0.60-2.40)	1.80 (1.10-2.80)	[18,20,21,35]
Ovary (C56)	Women	1.12 (0.90-1.39)	2.07 (1.65-2.60)	1.12 (0.90-1.39)^d^	2.07 (1.65-2.60)^d^	[22,29]
Kidney (C64)	Men	1.10 (0.90-1.20)	1.10 (0.90-1.20)	1.00 (0.70-1.40)	1.10 (0.80-1.50)	[21]
	Women	1.10 (0.60-2.10)	1.00 (0.60-1.60)	2.30 (0.90-6.30)	1.50 (0.60-3.90)	[21]
Bladder (C67)	Men	1.50 (1.30-1.70)	2.00 (1.70-2.30)	1.30 (0.90-1.90)	2.10 (1.40-2.90)	[21]
	Women	0.92 (0.53-1.58)	1.73 (1.26-2.38)	0.70 (0.20-2.20)	2.00 (1.10-3.80)	[21,32]

^a^RR for cancers of the oral cavity and pharynx combined was used; ^b^RR for ever-smoker (past + current smokers); ^c^RR for men was used for women; ^d^RR for incidence was used.

**Table 2:** Lung cancer cases and deaths among never-smokers attributable to passive smoking in Korea (2009)

	**Gender**	**Prevalence**^**a **^**(%) of passive smoking**	**RR**^**b **^**for lung cancer**	**PAF (%)**	**Lung cancer incidence cases/deaths among never-smokers**	**Passive smoking-related lung cancer cases/deaths**	**Sources of pooled RR**
Incidence							
Exposure to smoking at home	Men	14.8	1.00 (0.67-1.48)	-	1,109	0	[37,52,53]
	Women	60.1	1.32 (1.13-1.55)	16.3	4,809	783	[37-44,46,47,49-51,53,55-58]
Exposure to smoking at workplace	Men	42.2^c^	1.15 (0.74-1.77)	5.9	1,109	66	[52]
	Women	14.7^c^	1.37 (1.18-1.60)	5.2	4,809	251	[42,44,51,55-58]
Exposure to smoking at home or workplace	Men			5.9	1,109	66	
	Women			20.7	4,809	994	
% of all cancers	Men					0.1	
	Women					1.1	
Mortality							
Exposure to smoking at home	Men	14.8	1.34 (0.82-2.17)	4.8	544	26	[48]
	Women	60.1	1.32 (0.95-1.83)	16.1	3,543	571	[45,48]
Exposure to smoking at workplace^d^	Men	42.2^c^	1.15 (0.74-1.77)	5.9	544	32	[52]
	Women	14.7^c^	1.37 (1.18-1.60)	5.2	3,543	185	[42,44,51,55-58]
Exposure to smoking at home or workplace	Men			10.5	544	57	
	Women			20.5	3,543	726	
% of all cancers (aged 20+ years)	Men					0.1	
	Women					2.8	

^a^Prevalence of passive smoking at home or workplace was estimated by extrapolating the data from the Korea National Health and Nutrition Examination Survey in 2007, 2008, 2009, 2010, 2011 and 2012. [17].

^b^RRs obtained from a meta-analysis.

^c^Prevalence for passive smoking at the workplace in the Korean population was calculated by exposure prevalence at the workplace ×% employed adults in Korea in 1989: 71.2% in men, 45.7% in women (Statistics Korea) [60].

^d^RR for cancer incidence was used for cancer mortality.

**Figure 1:**
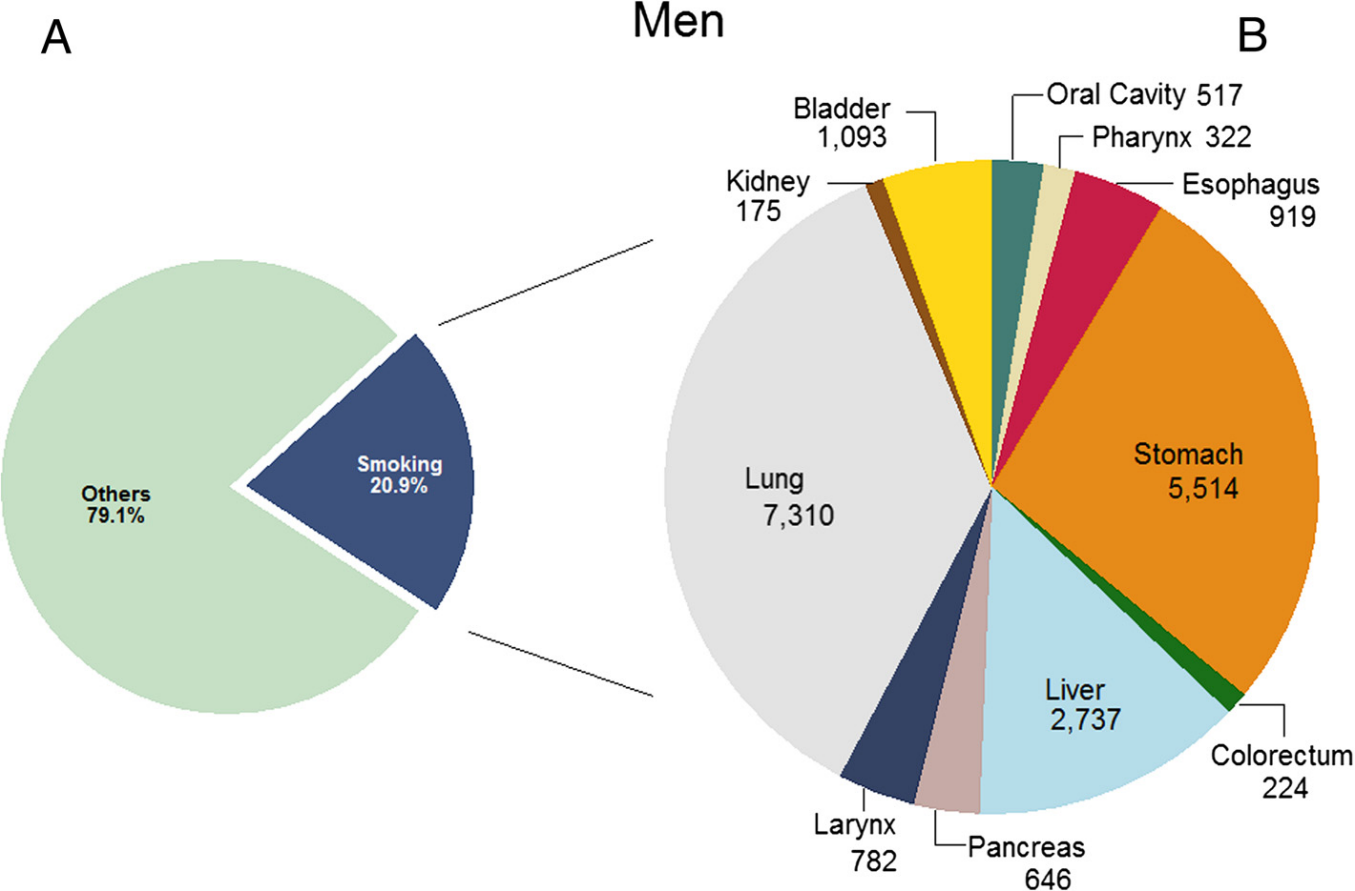
Number of cancer incident cases attributable to tobacco smoking in Korean men, 2009*. * **A)** Proportion of cancer incident cases attributable to tobacco smoking; **B)** Number of cancer incident cases attributable to tobacco smoking by cancer sites.

**Figure 2:**
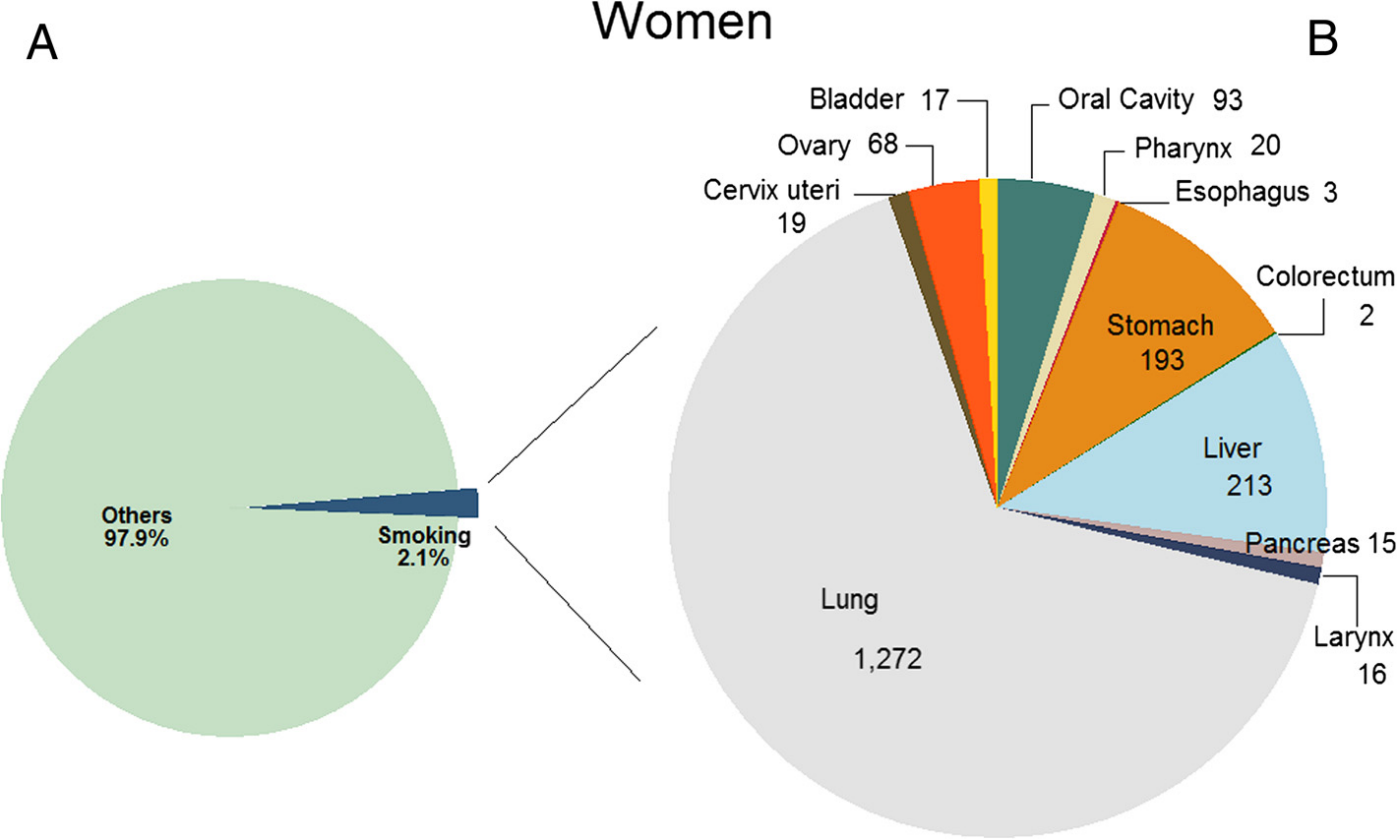
Number of cancer incident cases attributable to tobacco smoking in Korean women, 2009*. * **A)** Proportion of cancer incident cases attributable to tobacco smoking; **B)** Number of cancer incident cases attributable to tobacco smoking by cancer sites.

**Figure 3:**
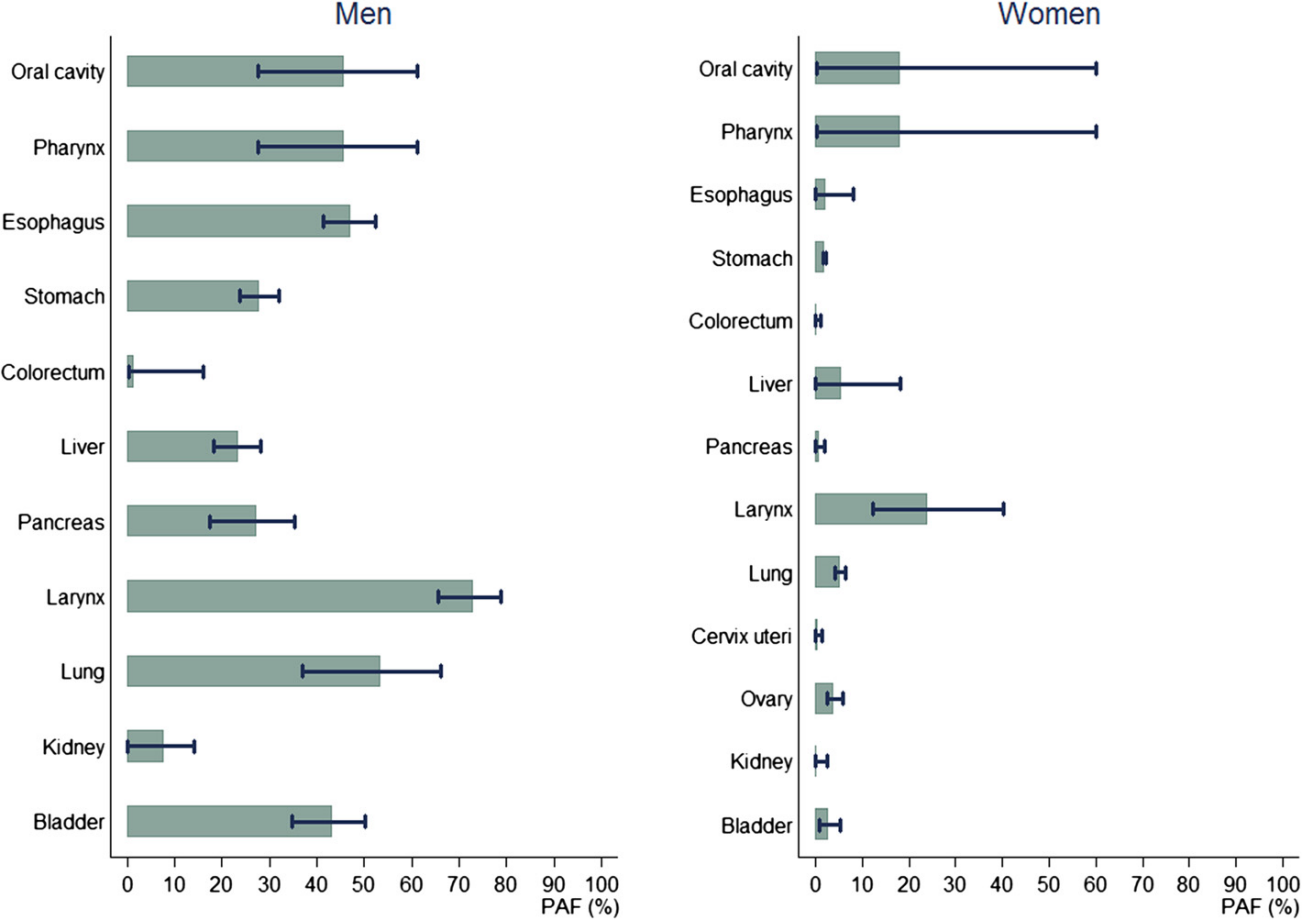
**Sensitivity analysis of the PAF for tobacco smoking using the lower and upper limits of 95% confidence interval for relative risks.** Note: the length of shaded bars represent the estimated PAF values when RRs in Table 1 were used and the intervals represent the PAF estimated using the lower and upper limits of 95% confidence interval for RRs.

Tobacco smoking was responsible for 14,377 (32.9%) cancer deaths among adult men and 1,351 (5.2%) cancer deaths among adult women in 2009 in Korea (Table 3). Overall, 11.8% of all adult cancer cases and 22.7% of all adult cancer deaths were attributable to either ever-smoking or second-hand tobacco smoking. In men, 71% of lung cancer deaths, 55%–72% of upper aerodigestive tract (oral cavity, pharynx, esophagus and larynx) cancer deaths, 23% of liver, 32% of stomach, 27% of pancreas, 7% of kidney and 45% of bladder cancer deaths were attributable to tobacco smoking. In women, however, ever-smoking-attributable lung cancer deaths were only 8.1% of the total lung cancer deaths. The PAF of second-hand smoking (20.5%) exceeded that of ever-smoking in Korean women because a large number of Korean women were exposed to second-hand smoking either at home (60%) or at workplace (15%), while ever-smoking among Korean women was not very prevalent (0.3% former smokers and 3.9% current smokers among Korean women in 1989). As expected, lung cancer comprised the greatest portion of all smoking-related cancer cases in men (36%, (7244 + 66)/20239) and women (66%, (278 + 994)/1930), followed by stomach and liver cancers (Figures 1 and 2).Sensitivity analysis showed that the PAF estimates were more sensitive to the variation in RR in women than in men when the upper and lower limits of the 95% CI of RR was used, due to the larger uncertainty in the estimation of RRs for women, particularly for oral cavity and pharynx cancer (Figure 3).

**Table 3:** Estimated number of cancer incidence cases and deaths attributable to tobacco smoking in Korea

**Cancer site**	**Men**				**Women**				**Total**			
	**PAF (%)**	**Cases**	**PAF (%)**	**Deaths**	**PAF (%)**	**Cases**	**PAF (%)**	**Deaths**	**PAF (%)**	**Cases**	**PAF (%)**	**Deaths**
Oral cavity	45.8	517	62.0	246	18.2	93	8.2	13	37.2	610	47.1	259
Pharynx	45.8	322	62.0	228	18.2	20	8.2	5	42.0	342	55.1	233
Esophagus	47.2	919	54.8	711	2.3	3	0.2	0	44.0	922	50.6	711
Stomach	27.9	5,514	31.6	2,107	2.0	193	0.2	5	19.4	5,707	20.8	2,112
Colorectum	1.5	224	1.2	45	0.0	1	0.4	13	0.9	225	0.8	58
Liver	23.5	2,737	23.5	1,976	5.5	213	6.1	172	19.0	2,950	19.1	2,148
Pancreas	27.4	646	26.8	598	0.8	15	0.4	7	15.5	661	14.9	605
Larynx	73.0	782	71.9	275	24.0	16	9.2	4	70.2	798	65.8	279
Lung	53.3	7,244	71.5	7,783	5.2	278	8.1	327	39.8	7,522	54.4	8,110
Among non-smokers	5.9	66	10.5	57	20.7	994	20.5	726	17.9	1,060	19.2	783
Cervix uteri					0.5	19	3.1	30	0.5	19	3.1	30
Ovary					4.0	68	4.0	34	4.0	68	4.0	34
Kidney	7.6	175	6.6	33	0.0	0	2.3	5	5.2	175	5.1	38
Bladder	43.4	1,093	44.9	318	2.8	17	3.8	10	35.4	1,110	34.0	328
Total	20.9	20,239	32.9	14,377	2.1	1,930	5.2	1,351	11.8	22,169	22.7	15,728
% of all cancers (aged 20+ years)		20.9		32.9		2.1		5.2		11.8		22.7

## Discussion

Our study provides a systematic assessment of the burden of smoking-related cancer in Korea in 2009. Overall, among 187,894 cancer incident cases in Korean adults in 2009, 22,169 (11.8%) were attributable to tobacco smoking. For cancer mortality, 15,728 of 69,431 (22.7%) cancer deaths were attributable to tobacco smoking in Korea. There was a large discrepancy between men and women in the PAF estimates of cancer incidence (20.9% vs. 2.1%) and cancer mortality (32.9% vs. 5.2%). Furthermore, the PAF of smoking was higher for cancer mortality than for cancer incidence. This is because smoking-related cancers, such as lung, liver, and pancreas cancer, tend to have a poor prognosis. Three in ten cancer deaths among Korean adult males in 2009 could have been prevented had there been no smokers in Korea. In particular, 71% of all lung cancer deaths in Korean adult males could have been prevented if no man had smoked in Korea.

The PAF of tobacco smoking for cancer mortality in Korean men was 33%, which was very similar to previous reports in France (33%), Japan (34%) and China (33%), and somewhat different from the UK (24%), a country in which smoking prevalence among men has decreased for several decades [5-8]. Interestingly, the overall cancer burden related to smoking in men in Korea is at about the same level as in France, Japan and China, although the RRs for current smokers are much lower in Korea, Japan and China than in France. It seems that the high smoking prevalence among Asian men adds to the smoking-attributable cases, while RRs have been observed to be lower in Asia than in Western countries. These results of comparison support the necessity of ethnic- or country-specific evaluation of the PAF because even though the overall PAF appear to be same, the exposure prevalences and the relative risks can be different across countries, therefore, the prevention strategy in each country should be also different. However, the PAF for Korean women appears to be much lower than France and UK, and somewhat lower than in China or Japan (Table 4). While the PAFs of lung cancer mortality for ever-smoking among men were very similar across three Asian countries, namely Korea, Japan and China, the PAFs among women were rather different. This seems to be due to the lower smoking prevalence and slightly lower RRs among Korean adult women compared to Japanese or Chinese women. Peto et al. estimated the cancer mortality attributable to smoking in 40 developed countries [65]. The estimates in the Central Asian population were 34% for men and 4% for women. Our Korean estimates were compatible with their figures.

**Table 4:** International comparison of PAF (%) of cancer deaths for tobacco smoking

**Cancer site (ICD-10 )**	**Republic of Korea**				**Japan **[7]				**China **[8]				**France **[5]				**United Kingdom **[6]			
	**Men**		**Women**		**Men**		**Women**		**Men**		**Women**		**Men**		**Women**		**Men**		**Women**	
	**RR**	**PAF**	**RR**	**PAF**	**RR**	**PAF**	**RR**	**PAF**	**RR**	**PAF**	**RR**	**PAF**	**RR**	**PAF**	**RR**	**PAF**	**RR**	**PAF**	**RR**	**PAF**
Oral Cavity (C00-C09)	3.3	62.0	3.3	8.2	2.4	50.0	1.8	8.5	1.5^b^	24.6	1.5^a^	2.8	4.2	63.1	1.6	17.0	10.9	70	5.1	55
Pharynx (C10-C14)													6.8	76.0	3.3	44.1				
Esophagus (C15)	2.6	54.8	0.9	0.2	3.0	58.9	2.4	14.7	1.3^c^	17.9	1.3^a^	1.9	2.5	51.1	2.3	34.4	6.8	63	7.8	71
Stomach (C16)	1.6	31.6	1.0	0.2	1.4	23.5	1.3	3.4	1.7^c^	30.9	1.7^a^	3.8	1.7	31.1	1.5	14.3	2.2	26	1.5	15
Colorectum (C18-C20)	1.1	1.2	1.1	0.4	1.4	20.4	1.4	4.5									1.24	7	1.3	10
Liver (C22)	1.4	23.5	2.6	6.1	1.7	35.1	1.6	6.8	1.4^b^	18.7	1.2^b^	1.0	1.9	37.5	1.5	17.1	2.3	27	1.5	15
Pancreas (C25)	1.5	26.8	1.1	0.4	1.4	23.9	1.9	9.5	1.9^c^	35.5	1.9^a^	4.6	1.6	24.9	1.6^d^	17.0	2.2	26	2.2	31
Larynx (C32)	4.5	71.9	3.6	9.2	4.5	71.9	4.5	30.1	1.5^b^	24.6	1.5^a^	2.8	5.2	75.9	5.2^d^	64.8	14.6	79	13	79
Lung (C33-C34)	4.4	71.5	3.2	8.1	3.6	67.5	3.6	23.9	5.7^c^	75.0	5.0^c^	18.4	9.9	83.0	7.6	69.2	21.3	87	12.5	84
Cervix (C53)	-	-	1.8	3.1	-	-	2.0	10.9	-	-	1.8^e^	4.5	-	-	1.8	22.9	-	-	1.5	7
Ovary (C56)	-	-	2.1	4.0	-	-	0.9	-									-	-	2.1	3
Kidney (C64)	1.1	6.6	1.1	2.3	1.5	27.9	0.9	-					1.6	26.4	1.4	11.5	2.5^g^	29^g^	1.5^g^	15^g^
Bladder (C67)	2.1	44.9	2.0	3.8	4.3^f^	70.7^f^	1.3^f^	3.6^f^	1.9^b^	36.8	1.7^b^	3.6	2.8	52.8	2.7	39.3	3	38	2.4	34
Myeloid leukemia (C92)	-	-	-	-	1.7	33.5	1.0	-									1.9	19	1.2	6
Prevalence (%)	70.8 (11.7)^h^		3.9 (0.3)^h^		53.1 (19.8)^i^		9.7 (2.6)^i^		64.0^j^		5.6^j^		48.2^k^		30.4^k^		22^l^		21^l^	
% of all cancers	32.9		5.2		34.4		6.2		32.7		5.0		33.4		9.6		23.0		15.6	

^a^Replaced by men’s RR.

^b^RR of cancer mortality associated with smoking. SE for RR (not 95% CI) is provided in Liu’s retrospective proportional mortality study (involving cancer of oral cavity, pharynx and larynx).

^c^RR of cancer incidence associated with smoking.

^d^When RRs for women were higher than for men or when no RR was estimable for women, the RR for men was used instead.

^e^RR not derived from Chinese studies.

^f^Renal pelvis, Ureter, Bladder (C65-68).

^g^Kidney and renal pelvis.

^h^Prevalence data for 1989 derived from the Korea National Health Examination Survey, current smoker’s prevalence and parenthesis represent former smoker’s prevalence.

^i^Prevalence data for 1990 derived from the National Nutrition Survey, current smoker’s prevalence and parenthesis represent former smoker’s prevalence.

^j^Prevalence data for ever-smoking and involuntary smoking of 1990 was estimated by linear interpolation using the results of these two national surveys.

^k^Prevalence data for 1985 were estimated by linear interpolation using results of surveys conducted in 1983 and 1986 (current smoker).

^l^Prevalence data for 2008 derived from General Lifestyle Survey 2008/ONS 2010 (current smoker).

The relative risks for smoking in Korea were much lower compared to those reported in Western countries, but rather similar to Japan or China (Table 4). This trend was particularly apparent in the RR for lung cancer, and the RR for kidney cancer was also relatively lower in Korea than other countries. The lung cancer risks observed among smokers in Asians are in general much lower than those in the Western population. A meta-analysis by Gandini et al. showed that smokers are at almost 10-fold elevated risk of developing lung cancer compared to never smokers in Caucasians, and 10-fold increase in African-Americans [66]. On the contrary, the lung cancer risk among current smokers is about four times the risk among never smokers in Asian countries such as Japan, China and Korea [67]. Furthermore, lung cancer rates in American men have greatly exceeded those in Japanese men for several decades despite the higher smoking prevalence in Japanese men, which was noted as “Japanese smoking paradox”. A multicentric case–control study involving both Americans and Japanese was carried out and showed a striking results that the odds ratio (OR) of current US smokers relative to never smokers was 40.4, that was about 6 to 10 times higher than the OR ranging between 3.5 and 6.3 in current Japanese smokers [68].

Epidemiologists have hypothesized that following may be possible explanations for these differences. First, many European countries and the U.S. began to experience their tobacco epidemic in 1920s, after World War I, approximately 30 years earlier than Asian countries [69]. In Korea, cigarette consumption has risen sharply since the end of Korean War in 1953, and the present rates of tobacco-caused disease in Asian countries should not be interpreted as reflecting lesser risks for smoking of Asian cigarettes. Second, ages at initiation of smoking are different. Korean smokers in all age groups started smoking later than their counterparts in Western countries, and they differed even more among female smokers [70]. Third, higher background risk of lung cancer among never-smokers is observed in Asians than individuals in Western countries. The lung cancer mortality rates among never smokers in Asian population (Rate = 35.6 in Japanese men; 24.6 in Japanese women) were indeed shown to be much higher than those in the US (Rate = 15.7 in CPS-I study; Rate = 14.7 in CPS-II study) [10,11,71].

According to a recent report on the mortality attributable to tobacco by World Health Organization, the estimated proportion of deaths from all malignant neoplasm attributable to tobacco was 35% for both sexes, 44% for men and 18% for women aged 30+ years in the Republic of Korea, which were higher than our estimates [72].

We also found that 20.7% of lung cancer cases among never smoking women were attributable to second-hand smoking from the home or workplace, and it is quite striking that the number of lung cancer incident cases (994 cases) related to second-hand smoking among never smoking women was about 3 times higher than that among smoking women (278 cases).

Our study has several strengths. First, we used nationwide cancer incidence and mortality data that achieve a nearly complete coverage of the Korean population. With well-established nationwide cancer and mortality registry systems, we had access to precise numbers of gender- and site-specific cancer cases and cancer deaths for PAF estimation. Second, the RR estimates for smoking and cancer used in our study were mostly derived from a very large-scale population-based cohort study with over 1,210,000 Korean subjects, giving reliable RR estimates that were also adjusted for confounding variables such as age and alcohol drinking. Therefore we believe that the potential bias of overestimating the PAF was minimized in our estimation. Third, the smoking prevalence was also obtained from national health survey data with a representative sample in 1989, which allowed for an induction period of 20 years.

Despite the strengths of our study, we acknowledge the limitations that might have resulted in underestimating smoking-attributable cancer fraction. A recent evaluation of smoking-related cancers also listed ureter and bone marrow cancers [2], but we did not include these because of lack of evidence of an increased RR in the Korean population. Furthermore, smoking in Korean women in 1989 might have been under-reported, because the survey was done through a personal interview, and smoking women were not culturally well-accepted, based on social norms in 1989 in Korea. Another limitation is that the restriction of our RR estimation to studies performed on Korean populations limited the number of studies included, which may have introduced slightly higher uncertainty in the pooled estimate of RRs. However, the PAFs were calculated from studies including a very large-scale population-based prospective study with over 1 million subjects, therefore we believe that the degree of uncertainty in our RR and PAF estimation was reduced.

## Conclusions

While the smoking prevalence in male adults has been decreasing in Korea, it remains among the highest of the developed countries. Because Korea is quickly approaching the status of an aged society, the number of cancer cases and deaths are expected to increase in the future. Furthermore, while lung cancer incidence rates have stabilized in men during recent years, those in women show a significantly increasing trend (annual percent change of 1.5%), which might reflect the fact that the smoking prevalence in women is increasing [59]. Approximately one out of three cancer deaths and two out of three lung cancer deaths in Korean men in 2009 could have been prevented had there been no smokers. And one out of four lung cancer cases among non-smoking Korean women could have been prevented if there had been no smokers. Considering the high prevalence of male smokers and increasing prevalence of young female smokers, effective control programs against tobacco smoking should be further developed and implemented in Korea to reduce the smoking-related cancer burden.

## Abbreviations

PAF: Population attributable fraction; CI: Confidence interval; RR: Relative risk; KNHES: Korea National Health Examination Survey.

## Competing interests

The authors declare that they have no competing interests.

## Authors’ contributions

HS and PB conceived of the study, and participated in its design and coordination and helped to draft the manuscript. EP participated in the literature search, performed the statistical analysis and helped to draft the manuscript. EC reviewed the literature on passive smoking and performed the meta-analysis. SJ and YY provided the re-analyzed data to estimate the pooled RRs. SH analyzed the data for the RR of passive smoking. SP participated in the design, literature search, statistical analysis, and wrote the manuscript. MB helped to apply various statistical analysis methods and to draft the manuscript. AS, KJ and SKP helped to draft the manuscript. All authors read and approved the final manuscript.

## Authors’ information

SP worked at the National Cancer Center until February 2012, and is now with the Graduate School of Public Health at Yonsei University. Aesun Shin worked at the National Cancer Center until August 2013, and is now with Seoul National University College of Medicine.

## Pre-publication history

The pre-publication history for this paper can be accessed here:

http://www.biomedcentral.com/1471-2407/14/406/prepub

## Supplementary Material

Additional file 1: Table S1 Studies included in the meta-analysis for tobacco smoking in Korean men. **Table S2.** Studies included in the meta-analysis for tobacco smoking in Korean women. **Table S3.** Studies included in the meta-analysis for passive smoking in men. **Table S4.** Studies included in the meta-analysis for passive smoking in women. **Table S5.** Estimation of cancer incident cases and deaths attributable to passive smoking among non-smokers. **Figure S1.** Prevalence (%) of tobacco smoking. **A)** Never smoker, **B)** Former smoker, **C)** Current smoker. **Figure S2.** Prevalence (%) of passive smoking **A)** At home, **B)** At workplace. **Figure S3.** Meta-analysis on passive smoking at home and lung cancer incidence in men. **Figure S4.** Meta-analysis on passive smoking at home and lung cancer incidence in women. **Figure S5.** Meta-analysis on passive smoking at home and lung cancer mortality in women. **Figure S6.** Meta-analysis on passive smoking at workplace and lung cancer incidence in wome. Click here for file
