# Supplementary material for: Attributable fraction of tobacco smoking on cancer using population-based nationwide cancer incidence and mortality data in Korea
Source: BMC Cancer. 2014 Jun 6;14:406. doi: 10.1186/1471-2407-14-406 (PMC4090397; doi:10.1186/1471-2407-14-406)
Supplement: Additional file 1: Table S1 — Studies included in the meta-analysis for tobacco smoking in Korean men. Table S2. Studies included in the meta-analysis for tobacco smoking in Korean women. Table S3. Studies included in the meta-analysis for passive smoking in men. Table S4. Studies included in the meta-analysis for passive smoking in women. Table S5. Estimation of cancer incident cases and deaths attributable to passive smoking among non-smokers. Figure S1. Prevalence (%) of tobacco smoking. A) Never smoker, B) Former smoker, C) Current smoker. Figure S2. Prevalence (%) of passive smoking A) At home, B) At workplace. Figure S3. Meta-analysis on passive smoking at home and lung cancer incidence in men. Figure S4. Meta-analysis on passive smoking at home and lung cancer incidence in women. Figure S5. Meta-analysis on passive smoking at home and lung cancer mortality in women. Figure S6. Meta-analysis on passive smoking at workplace and lung cancer incidence in wome. [file 1471-2407-14-406-S1.pdf]

Supplementary Table 1. Studies included in the meta-analysis for tobacco smoking in Korean men

Author (year)	Study period	Study subjects					Category of smoking	OR (95% CI)	Confounding variables considered
		Type and source	Definition	Cancer site	No. of cases	No. of controls			
Incidence									
Choi SY et al. (1992) [20]	1986-1990	Hospital-based (Korea Cancer Center Hospital)	Cases: newly diagnosed histologically cases<br><br>Controls: cancer-free patients at the same hospital	Oral cavity & Pharynx*			Never<br>Former<br>Current	1.00<br>0.87 (0.45-1.66)<br>2.01 (1.30-3.10)	
				Esophagus<br><br>Mean age	139<br><br>57.2	417<br><br>57.6	Never<br>Former<br>Current	1.00<br>1.11 (0.49-2.53)<br>1.93 (1.12-2.84)	Adjusted for age, marital status, education and alcohol use
				Stomach<br><br>Mean age	238<br><br>50.8	714<br><br>49.7	Never<br>Former<br>Current	1.00<br>0.89 (0.48-1.65)<br>1.34 (0.88-2.03)	Adjusted for age, marital status, education, diet and alcohol use
				Rectum<br><br>Mean age	67<br><br>52.6	201<br><br>52.4	Never<br>Former<br>Current	1.00<br>1.35 (0.53-2.28)<br>0.71 (0.35-1.45)	Adjusted for age, marital status, education, diet and alcohol use
				Larynx<br><br>Mean age	94<br><br>58.4	282<br><br>58.8	Never<br>Former<br>Current	1.00<br>2.24 (0.60-8.43)<br>5.41 (2.06-14.27)	Adjusted for alcohol use
				Lung<br><br>Mean age	280<br><br>55	840<br><br>53.3	Never<br>Former<br>Current	1.00<br>2.06 (0.98-4.31)<br>5.78 (3.07-10.89)	Adjusted for alcohol use
Jee SH et al. (2004) [21]<br>*Re-analysis using updated data*	1993-2001	National Health Insurance Corporation (NHIC)	Cohort<br>758,193 men	Oral cavity & Pharynx**<br><br>30 ≤ age ≤95	12<br>17<br>77		Never<br>Former<br>Current	1.0<br>1.3 (0.6-2.7)<br>2.6 (1.4-4.8)	Adjusted for age and alcohol
			Cohort<br>758,193 men	Esophagus**<br><br>30 ≤ age ≤95	155<br>220<br>1,045		Never<br>Former<br>Current	1.0<br>1.2 (0.9-1.4)<br>2.3 (2.0-2.8)	Adjusted for age and alcohol
			Cohort<br>758,193 men	Stomach**<br><br>30 ≤ age ≤95	2,184<br>3,046<br>8,850		Never<br>Former<br>Current	1.0<br>1.3 (1.2-1.4)<br>1.5 (1.5-16)	Adjusted for age, alcohol and diet

* From meta-analysis on oral cavity and pharynx.
** Additional analysis results on RR from updated dataset were obtained through personal communication with the author

Supplementary Table 1. Studies included in the meta-analysis for tobacco smoking in Korean men (continued)

Author (year)	Study period	Study subjects					Category of smoking	OR (95% CI)	Confounding variables considered
		Type and source	Definition	Cancer site	No. of cases	No. of controls			
Jee SH et al. (2004) [21] *Re-analysis using updated data*	1993-2001	National Health Insurance Corporation (NHIC)	Cohort<br>830,139 men	Colon<br><br>30 ≤ age ≤95	342<br>509<br>782		Never<br>Former<br>Current	1.0<br>1.1 (1.0-1.3)<br>0.8 (0.7-1.0)	Adjusted for age
			Cohort<br>441,883 men	Liver*<br><br>30 ≤ age ≤95	963<br>1,131<br>3,520		Never<br>Former<br>Current	1.0<br>1.2 (1.1-1.3)<br>1.4 (1.3-1.5)	Adjusted for age, alcohol and HBV
			Cohort<br>758,193 men	Pancreas*<br><br>30 ≤ age ≤95	299<br>385<br>1,126		Never<br>Former<br>Current	1.0<br>1.2 (1.0-1.4)<br>1.5 (1.3-1.7)	Adjusted for age and alcohol
			Cohort<br>758,193 men	Larynx*<br><br>30 ≤ age ≤95	58<br>137<br>722		Never<br>Former<br>Current	1.0<br>2.0 (1.5-2.8)<br>4.6 (3.6-6.1)	Adjusted for age and alcohol
			Cohort<br>758,193 men	Lung*<br><br>30 ≤ age ≤95	758<br>1,332<br>6,657		Never<br>Former<br>Current	1.0<br>1.6 (1.4-1.7)<br>3.7 (3.4-4.0)	Adjusted for age and alcohol
			Cohort<br>758,193 men	Kidney*<br><br>30 ≤ age ≤95	270<br>302<br>769		Never<br>Former<br>Current	1.0<br>1.1 (0.9-1.2)<br>1.1 (0.9-1.2)	Adjusted for age and alcohol
			Cohort<br>758,193 men	Bladder*<br><br>30 ≤ age ≤95	275<br>442<br>1,348		Never<br>Former<br>Current	1.0<br>1.5 (1.3-1.7)<br>2.0 (1.7-2.3)	Adjusted for age and alcohol
Kimm H et al. (2010) [26]	1993-2006	Korean Cancer Prevention Study (KCPS)	Cohort<br>782,632 men	Esophagus<br><br>30 ≤ age ≤93	150<br>224<br>1,009		Never<br>Former<br>Current	1.0<br>1.2 (1.0-1.4)<br>2.2 (1.8-2.5)	Adjusted for age, age^2, alcohol intake, aspartate aminotransferase(GOT), body mass index and exercise

* Additional analysis results on RR from updated dataset were obtained through personal communication with the author

Supplementary Table 1. Studies included in the meta-analysis for tobacco smoking in Korean men (continued)

Author (year)	Study period	Study subjects					Category of smoking	OR (95% CI)	Confounding variables considered
		Type and source	Definition	Cancer site	No. of cases	No. of controls			
Kim J et al. (2012) [23]	2003-2007	Hospital-based (National Cancer Center)	Cases: patients with noncardia gastric cancer<br><br>Controls: healthy controls who underwent upper endoscopy for gastric cancer screening and were without significant gastrointestinal symptoms	Stomach<br><br><br><br>Mean age	183<br>108<br>204<br><br>54.9±8.4	208<br>158<br>129<br><br>54.3±7.4	Never<br>Former<br>Current	1.00<br>0.78 (0.56-1.08)<br>1.80 (1.32-2.45)	Matched by age(±5) and sex
Yang JJ et al. (2009) [34]	2002	Korean Multi-Center Cancer Cohort (KMCC)	Cases: patients signed a consent form and completed a detailed standardized interview-based questionnaire	Stomach<br><br>40 ≤ age ≤83	26<br>21<br>37	141<br>82<br>111	Never<br>Former<br>Current	1.0<br>1.4 (0.8-2.6)<br>1.8 (1.0-3.2)	Matched by age(±5), sex, residential district and enrollment year<br><br>Adjusted for H.pylori infection, CagA seropositivity, age and sex
Lee JK et al. (1995) [28]	1990-1991	Hospital-based (Hanyang University Hospital, Asan Medical Center)	Cases: patients hospitalized in the surgical wards of two teaching hospitals<br><br>Controls: hospitalized patients with wide range of problems	Stomach<br><br>25 ≤ age ≤65	82<br>49<br>82	107<br>42<br>63	Never<br>Former<br>Current	1.0<br>3.0 (1.4-6.2)<br>3.4 (1.7-6.8)	Matched by age(±2) and sex<br><br>Adjusted for age, sex, education, economic status and residence

Supplementary Table 1. Studies included in the meta-analysis for tobacco smoking in Korean men (continued)

Author (year)	Study period	Study subjects					Category of smoking	OR (95% CI)	Confounding variables considered
		Type and source	Definition	Cancer site	No. of cases	No. of controls			
Kim J et al. (2009) [24]	2001-2004	Hospital-based (Two university hospitals)	Cases: reconfirmed from both pathology report and chart review<br><br>Controls: had been hospitalized during the same period for a wide spectrum of non-neoplastic conditions	Colorectum<br><br>30 ≤ age ≤79	86<br>100<br>176	84<br>80<br>106	Never<br>Former<br>Current	1.00<br>1.22 (0.78-1.90)<br>1.62 (1.08-2.43)	Matched by age
Shin A et al. (2011) [33]	1996-1997	Korean national Health System (KNHS)	Cohort<br>869,725 men	Colorectum*<br><br>30 ≤ age ≤80			Never<br>Former<br>Current	1.00<br>1.18 (0.96-1.44)<br>0.98 (0.90-1.07)	
Park JY et al. (2002) [30]	1997-2000	Hospital-based (Kyungpook National University Hospital)	Cases: diagnosed with lung cancer<br><br>Controls: randomly selected from a pool of healthy volunteers	Lung<br><br>Mean age	2<br>6<br>184<br><br>61.2±8.4	3<br>37<br>95<br><br>60.7±8.4	Never<br>Former<br>Current	1.00<br>0.24 (0.02-3.62)<br>2.91 (0.33-35.19)	Matched by age(±5)
Choi JE et al. (2009) [19]	2001-2002	Hospital-based (Kyungpook National University Hospital)	Cases: lung cancer patients<br><br>Controls: randomly selected from a pool of healthy volunteers	Lung<br><br>Mean age	141<br>116<br>463<br><br>61.3±8.8	198<br>191<br>331<br><br>60.6±9.3	Never<br>Former<br>Current	1.00<br>0.85 (0.61-1.18)<br>1.96 (1.51-2.56)	Matched by age(±5) and sex
Kim JS et al. (2010) [25]	2001-2004	Hospital-based (Kyungpook National University Hospital)	Cases: lung cancer patients<br><br>Controls: healthy volunteers who visited the general health check-up center	Lung**<br><br>Mean age:<br>Stage1<br>Stage2	 <br><br>61.5±10.9<br>60.9±8.5	 <br><br>61.5±11.3<br>60.5±9.0	Never<br>Former<br>Current	1.00<br>0.51 (0.12-2.09)<br>1.83 (1.37-2.44)	

* Meta-analysis of RRs for proximal colon, distal colon, and rectum
** Meta-analysis of R

Supplementary Table 1. Studies included in the meta-analysis for tobacco smoking in Korean men (continued)

Author (year)	Study period	Study subjects					Category of smoking	OR (95% CI)	Confounding variables considered
		Type and source	Definition	Cancer site	No. of cases	No. of controls			
Yoon KA et al. (2007) [36]	2002-2003	Hospital-based (National Cancer Center)	Cases: patients with histologically confirmed lung cancer<br><br>Controls: without a prior history of cancer were recruited form the visitors for cancer-screening program	Lung<br><br>age range	116<br>75<br>143<br>25≤age≤70	139<br>103<br>86<br>28≤age≤73	Never<br>Former<br>Current	1.00<br>0.87 (0.58-1.31)<br>1.99 (1.36-2.91)	Matched by age(±3) and sex
Park SK et al. * (2010) [31]<br>Re-analysis	1997-1998	Korean Academy of Tuberculosis and Respiratory Diseases (KATRD)<br>Korean Multi-Center Cancer Cohort (KMCC)	Cases: histologically confirmed incident lung cancer cases diagnosed in 1997 were registered in the KATRD<br><br>Controls: cancer free when registered in 1997 in Chungju, a site of the KMCC	Lung<br><br>44 ≤ age ≤70	104<br>158<br>619	101<br>90<br>261	Never<br>Former<br>Current	1.00<br>1.72 (1.18-2.51)<br>2.31 (1.70-3.16)	Matched by age(≤44, 45-69, and ≥70 years old)<br><br>Adjusted for age
Mortality									
Jee SH et al. (2004) [21]<br>*Re-analysis using updated data*	1993-2001	National Health Insurance Corporation (NHIC)	Cohort<br>758,193 men	Oral cavity & Pharynx**<br><br>30 ≤ age ≤95	1<br>1<br>10		Never<br>Former<br>Current	1.0<br>0.8 (0.1-13.5)<br>3.3 (0.5-34.6)	Adjusted for age and alcohol
			Cohort<br>758,193 men	Esophagus**<br><br>30 ≤ age ≤95	80<br>140<br>630		Never<br>Former<br>Current	1.0<br>1.4 (1.1-1.8)<br>2.8 (2.2-3.5)	Adjusted for age and alcohol
			Cohort<br>758,193 men	Stomach**<br><br>30 ≤ age ≤95	830<br>1,201<br>3,305		Never<br>Former<br>Current	1.0<br>1.3 (1.2-1.4)<br>1.6 (1.5-1.7)	Adjusted for age, alcohol and diet
			Cohort<br>830,139 men	Colon<br><br>30 ≤ age ≤95	91<br>139<br>281		Never<br>Former<br>Current	1.0<br>1.1 (0.9-1.4)<br>1.1 (0.8-1.4)	Adjusted for age

* Additional analysis results on RR from dataset were obtained through personal communication with the author
** Additional analysis results on RR from updated dataset were obtained through personal communication with the author

Supplementary Table 1. Studies included in the meta-analysis for tobacco smoking in Korean men (continued)

Author (year)	Study period	Study subjects					Category of smoking	OR (95% CI)	Confounding variables considered
		Type and source	Definition	Cancer site	No. of cases	No. of controls			
Jee SH et al. (2004) [21]<br>*Re-analysis using updated data*	1993-2001	National Health Insurance Corporation (NHIC)	Cohort<br>441,883 men	Liver*<br><br>30 ≤ age ≤95	573<br>672<br>2,162		Never<br>Former<br>Current	1.0<br>1.2 (1.0-1.3)<br>1.4 (1.3-1.6)	Adjusted for age, alcohol and HBV
			Cohort<br>758,193 men	Pancreas*<br><br>30 ≤ age ≤95	221<br>281<br>816		Never<br>Former<br>Current	1.0<br>1.1 (0.9-1.3)<br>1.5 (1.3-1.7)	Adjusted for age and alcohol
			Cohort<br>758,193 men	Larynx*<br><br>30 ≤ age ≤95	20<br>41<br>228		Never<br>Former<br>Current	1.0<br>1.7 (1.0-2.9)<br>4.5 (2.8-7.1)	Adjusted for age and alcohol
			Cohort<br>758,193 men	Lung*<br><br>30 ≤ age ≤95	423<br>871<br>4,381		Never<br>Former<br>Current	1.0<br>1.8 (1.6-2.0)<br>4.4 (4.0-4.9)	Adjusted for age and alcohol
			Cohort<br>758,193 men	Kidney*<br><br>30 ≤ age ≤95	52<br>58<br>146		Never<br>Former<br>Current	1.0<br>1.0 (0.7-1.4)<br>1.1 (0.8-1.5)	Adjusted for age and alcohol
			Cohort<br>758,193 men	Bladder*<br><br>30 ≤ age ≤95	39<br>58<br>176		Never<br>Former<br>Current	1.0<br>1.3 (0.9-1.9)<br>2.1 (1.4-2.9)	Adjusted for age and alcohol
Kimm H et al. (2010) [26]	1993-2006	Korean Cancer Prevention Study (KCPS)	Cohort<br>782,632 men	Esophagus<br><br>30 ≤ age ≤93	92<br>183<br>721		Never<br>Former<br>Current	1.0<br>1.5 (1.2-2.0)<br>2.5 (2.0-3.1)	Adjusted for age, age^2, alcohol intake, aspartate aminotransferase(GOT), body mass index and exercise

* Additional analysis results on RR from updated dataset were obtained through personal communication with the author

Supplementary Table 1. Studies included in the meta-analysis for tobacco smoking in Korean men (continued)

Author (year)	Study period	Study subjects					Category of smoking	OR (95% CI)	Confounding variables considered
		Type and source	Definition	Cancer site	No. of cases	No. of controls			
Lee EH et al. * (2010) [27] Re-analysis	1993-2004	Korean Multi-Center Cancer Cohort (KMCC)	Cohort<br>5,287 men	Stomach<br><br>40 ≤ age	6<br>22<br>42		Never<br>Former<br>Current	1.00<br>3.10 (1.25-7.66)<br>2.41 (1.02-5.70)	Adjusted for age, body mass index and alcohol consumption amount
			Cohort<br>5,287 men	Pancreas<br><br>40 ≤ age	2<br>6<br>6		Never<br>Former<br>Current	1.00<br>2.44 (0.49-12.26)<br>1.21 (0.24-6.12)	Adjusted for age, body mass index and alcohol consumption amount
			Cohort<br>5,287 men	Lung<br><br>40 ≤ age	5<br>21<br>62		Never<br>Former<br>Current	1.00<br>3.54 (1.33-9.41)<br>4.48 (1.80-11.19)	Adjusted for age, body mass index and alcohol consumption amount

* Additional analysis results on RR from dataset were obtained through personal communication with the author

Supplementary Table 2. Studies included in the meta-analysis for tobacco smoking in Korean women

Author (year)	Study period	Study subjects					Category of smoking	RR (95% CI)	Confounding variables considered
		Type and source	Definition	Cancer site	No. of cases	No. of controls			
Incidence									
Choi SY et al. (1992) [20]	1986-1990	Hospital-based (Korea Cancer Center Hospital)	Cases: newly diagnosed and histologically confirmed cases<br><br>Controls: cancer-free patients at the same hospital	Cervix<br><br>Mean age	271<br><br>46.8	813<br><br>46.6	Never<br>Former<br>Current	1.00<br>2.52 (0.84-7.62)<br>0.77 (0.45-1.31)	Adjusted for marital status and alcohol use
Jee SH et al. * (2004) [21]<br>*Re-analysis using updated data*	1993-2001	National Health Insurance Corporation (NHIC)	Cohort<br>434,145 women	Oral cavity & Pharynx<br><br>30 ≤ age ≤95	8<br>0<br>2		Never<br>Former<br>Current	1.0<br>-<br>6.7 (1.1-39.4)	Adjusted for age and alcohol
			Cohort<br>434,145 women	Esophagus<br><br>30 ≤ age ≤95	69<br>4<br>11		Never<br>Former<br>Current	1.0<br>1.1 (0.4-3.1)<br>1.6 (0.8-3.1)	Adjusted for age and alcohol
			Cohort<br>121,822 women	Liver<br><br>30 ≤ age ≤95	223<br>1<br>5		Never<br>Former<br>Current	1.0<br>0.8 (0.1-5.6)<br>2.5 (1.0-6.3)	Adjusted for age, alcohol and HBV
			Cohort<br>434,145 women	Pancreas<br><br>30 ≤ age ≤95	678<br>27<br>75		Never<br>Former<br>Current	1.0<br>0.8 (0.5-1.1)<br>1.2 (0.9-1.5)	Adjusted for age and alcohol
			Cohort<br>434,145 women	Larynx<br><br>30 ≤ age ≤95	28<br>1<br>19		Never<br>Former<br>Current	1.0<br>0.9 (0.1-6.8)<br>9.1 (4.6-17.8)	Adjusted for age and alcohol
			Cohort<br>434,145 women	Lung<br><br>30 ≤ age ≤95	1,709<br>119<br>317		Never<br>Former<br>Current	1.0<br>1.6 (1.3-1.9)<br>2.3 (2.0-2.6)	Adjusted for age and alcohol
			Cohort<br>434,145 women	Cervix<br><br>30 ≤ age ≤95	2,117<br>42<br>100		Never<br>Former<br>Current	1.0<br>1.0 (0.7-1.3)<br>1.1 (0.9-1.4)	Adjusted for age and alcohol

* Additional analysis results on RR from updated dataset were obtained through personal communication with the author

Supplementary Table 2. Studies included in the meta-analysis for tobacco smoking in Korean women (continued)

Author (year)	Study period	Study subjects					Category of smoking	RR (95% CI)	Confounding variables considered
		Type and source	Definition	Cancer site	No. of cases	No. of controls			
Jee SH et al. * (2004) [21] *Re-analysis using updated data*	1993-2001	National Health Insurance Corporation (NHIC)	Cohort<br>434,145 women	Kidney<br><br>30 ≤ age ≤95	307<br>10<br>16		Never<br>Former<br>Current	1.0<br>1.1 (0.6-2.1)<br>1.0 (0.6-1.6)	Adjusted for age and alcohol
			Cohort<br>434,145 women	Bladder<br><br>30 ≤ age ≤95	253<br>9<br>35		Never<br>Former<br>Current	1.0<br>0.8 (0.4-1.7)<br>1.8 (1.2-2.6)	Adjusted for age and alcohol
Kim J et al. (2009) [24]	2001-2004	Hospital-based (Two university hospitals)	Cases: reconfirmed from both pathology report and chart review<br><br>Controls: had been hospitalized during the same period for a wide spectrum of non-neoplastic conditions	Colorectum<br><br><br>30 ≤ age ≤79	216<br>12<br>7	229<br>6<br>4	Never<br>Former<br>Current	1.00<br>2.12 (0.72-7.00)<br>1.86 (0.46-8.75)	Matched by age
Shin A et al. (2011) [33]	1996-1997	Korean national Health System (KNHS)	Cohort<br>395,501 women	Colorectum**<br><br><br>30 ≤ age ≤80			Never<br>Former<br>Current	1.00<br>0.96 (0.61-1.51)<br>0.96 (0.75-1.23)	
Park SK et al. * (2010) [31] Re-analysis	1997-1998	Korean Academy of Tuberculosis and Respiratory Diseases (KATRD)<br>Korean Multi-Center Cancer Cohort (KMCC)	Cases: histologically confirmed incident lung cancer cases diagnosed in 1997 were registered in the KATRD<br><br>Controls: cancer free when registered in 1997 in Chungju, a site of the KMCC	Lung<br><br><br>44 ≤ age ≤70	499<br>20<br>101	564<br>8<br>39	Never<br>Former<br>Current	1.00<br>2.96 (1.29-6.81)<br>3.06 (2.06-4.55)	Matched by age(≤44, 45-69, and ≥70 years old)<br><br>Adjusted for age

* Additional analysis results on RR from updated dataset were obtained through personal communication with the author
** Meta-analysis of RRs for proximal colon, distal colon, and rectum

Supplementary Table 2. Studies included in the meta-analysis for tobacco smoking in Korean women (continued)

Author (year)	Study period	Study subjects					Category of smoking	RR (95% CI)	Confounding variables considered
		Type and source	Definition	Cancer site	No. of cases	No. of controls			
Cho H et al. (2009) [18]	2006-2007	Hospital-based (six academic medical centers)	Cases: invasive cervical cancer patients<br><br>Controls: healthy women who had no history of an abnormal Pap smear and normal Pap smear on the day of recruitment	Cervix<br><br><br><br>Mean age	140<br>6<br>14<br><br>51.5±12.2	343<br>12<br>23<br><br>46.8±10.1	Never<br>Former<br>Current	1.00<br>1.23 (0.37-3.61)<br>1.49 (0.69-3.12)	
Yoo KY et al.* (1997) [35] *Re-analysis using updated data*	1992-1995	Seoul National University Hospital	Cases: Histologically confirmed cases of invasive cervical cancer were selected from the Department of Gynecology, Seoul National University Hospital<br><br>Controls: Women with normal pap smear tests and women free of past history of any Malignancies	Cervix	167<br>7<br>10	738<br>8<br>22	Never<br>Former<br>Current	1.00<br>2.78 (0.93-8.36)<br>2.21 (0.99-4.95)	
Ma SH et al. (2012) [29] *Re-analysis using updated data*	2009-ongoing	Korea Epithelial Ovarian Cancer Study (Ko-Eve)	Cases: newly diagnosed histologically epithelial ovarian cancer cases<br><br>Controls: ovarian cancer-free population lived in the community	Ovary	892<br>7<br>2	165<br>5<br>3	Never<br>Former<br>Current	1.00<br>2.68 (0.49-14.56)<br>0.96 (0.18-5.02)	Adjusted for all variables

* Additional analysis results on RR from dataset were obtained through personal communication with the author

Supplementary Table 2. Studies included in the meta-analysis for tobacco smoking in Korean women (continued)

Author (year)	Study period	Study subjects					Category of smoking	RR (95% CI)	Confounding variables considered
		Type and source	Definition	Cancer site	No. of cases	No. of controls			
Jordan et al. (2006) [22]	1966-2005	Systematic literature review	Eight population-based case-control studies	Ovary			Never<br>Former<br>Current	1.0<br>1.0 (0.9-1.2)<br>2.1 (1.7-2.7)	
Seo HS et al. (2005) [32]	1993-2002	Korean Medical Insurance Corporation (KMIC)'s dependents	Cohort<br>263,915 women	Bladder<br><br>Mean age<br>Never: 54.4±9.6<br>Former: 64.3±8.6<br>Current: 62.2±8.6			Never<br>Former<br>Current	1.0<br>1.1 (0.5-2.6)<br>1.6 (0.9-2.7)	Adjusted for age, sex, Rohrer's index, blood pressure, cholesterol level, blood glucose level, blood pigment, hypertension, diabetes etc.
Mortality									
Jee SH et al. * (2004) [21]<br>*Re-analysis using updated data*	1993-2001	National Health Insurance Corporation (NHIC)	Cohort<br>434,145 women	Esophagus<br><br>30 ≤ age ≤95	28<br>4<br>4		Never<br>Former<br>Current	1.0<br>1.6 (0.6-5.1)<br>0.9 (0.3-2.7)	Adjusted for age and alcohol
			Cohort<br>434,145 women	Stomach<br><br>30 ≤ age ≤95	1,322<br>77<br>140		Never<br>Former<br>Current	1.0<br>1.0 (0.8-1.2)<br>1.0 (0.8-1.2)	Adjusted for age, alcohol and diet
			Cohort<br>121,822 women	Liver<br><br>30 ≤ age ≤95	84<br>1<br>2		Never<br>Former<br>Current	1.0<br>1.9 (0.3-14.2)<br>2.6 (0.6-11.0)	Adjusted for age, alcohol and HBV
			Cohort<br>434,145 women	Pancreas<br><br>30 ≤ age ≤95	503<br>25<br>57		Never<br>Former<br>Current	1.0<br>0.9 (0.5-1.2)<br>1.1 (0.8-1.4)	Adjusted for age and alcohol
			Cohort<br>434,145 women	Larynx<br><br>30 ≤ age ≤95	13<br>1<br>7		Never<br>Former<br>Current	1.0<br>0.9 (0.1-6.9)<br>3.6 (1.3-9.7)	Adjusted for age and alcohol
			Cohort<br>434,145 women	Lung<br><br>30 ≤ age ≤95	834<br>79<br>234		Never<br>Former<br>Current	1.0<br>1.9 (1.5-2.4)<br>3.2 (2.7-3.7)	Adjusted for age and alcohol

* Additional analysis results on RR from updated dataset were obtained through personal communication with the author

Supplementary Table 2. Studies included in the meta-analysis for tobacco smoking in Korean women (continued)

Author (year)	Study period	Study subjects					Category of smoking	RR (95% CI)	Confounding variables considered
		Type and source	Definition	Cancer site	No. of cases	No. of controls			
Jee SH et al. * (2004) [21] *Re-analysis using updated data*	1993-2001	National Health Insurance Corporation (NHIC)	Cohort<br>434,145 women	Cervix<br><br>30 ≤ age ≤95	176<br>9<br>24		Never<br>Former<br>Current	1.0<br>1.2 (0.6-2.4)<br>1.8 (1.1-2.8)	Adjusted for age and alcohol
			Cohort<br>434,145 women	Kidney<br><br>30 ≤ age ≤95	37<br>5<br>6		Never<br>Former<br>Current	1.0<br>2.3 (0.9-6.3)<br>1.5 (0.6-3.9)	Adjusted for age and alcohol
			Cohort<br>434,145 women	Bladder<br><br>30 ≤ age ≤95	48<br>3<br>15		Never<br>Former<br>Current	1.0<br>0.7 (0.2-2.2)<br>2.0 (1.1-3.8)	Adjusted for age and alcohol
Lee EH et al. * (2010) [27] Re-analysis	1993-2004	Korean Multi-Center Cancer Cohort (KMCC)	Cohort<br>8,874women	Stomach<br><br>40 ≤ age	14<br>1<br>5		Never<br>Former<br>Current	1.00<br>2.85 (0.36-	Adjusted for age, body mass index and alcohol consumption amount

* Additional analysis results on RR from updated dataset were obtained through personal communication with the author

Supplementary Table 3. Studies included in the meta-analysis for passive smoking in men

Cancer site	Author (year)	Study period	Study subjects				OR (95% CI)	Confounding variables considered
			Type and source	Definition	No. of cases	No. of controls		
Lung	***Exposure to smoking at home, Incidence***							
	Tse LA et al. (2009) [52]	2004-2006	Population-based case-referent	Cases: nonsmoker with newly diagnosed primary lung cancer<br>Controls: nonsmoking community referents	132	536	0.90 (0.57-1.41)	Adjusted for age, place of birth, alcohol drinking, residential radon exposure, past history of lung diseases, any cancer in first-degree relatives, intakes of meat, exposure to known or suspected lung carcinogens, and adoption of dust control
	Akiba S et al. (1986) [37]	1971-1980	Radiation Effects Research Foundation (RERF)	Cases: non-smoking men with newly diagnosed cases of primary lung cancer among cohort members<br>Controls: non-smoking men among cohort members without lung cancer	19	110	2.10 (0.33-9.63)	Individually matched for year of birth, city of residence, sex, and whether or not they were among the 20% of the cohort participating in the program of biennial medical examinations given at RERF, and vital status.
	Wang L et al. (2000) [53]	1994-1998	Hospital-based with community control	Cases: diagnosed lung cancer among never-smokers<br>Controls: randomly selected from 1990 census lists<br>30 ≤ age ≤75	33	114	1.22 (0.5-3.3)	Frequency matched for age, sex, and prefecture of current residence
	Exposure to smoking at home, Mortality							
	McGhee SM et al. (2005) [48]	1998	Death registries	Cases: death from lung cancer among never smoker<br>Controls: never smoking controls	145	418	1.34 (0.82-2.17)	Adjusted for age, education
	Exposure to smoking at workplace, Incidence							
	Tse LA et al. (2009) [52]	2004-2006	Population-based case-referent	Cases: nonsmoker with newly diagnosed primary lung cancer<br>Controls: nonsmoking community referents	132	536	1.15 (0.74-1.77)	Adjusted for age, place of birth, alcohol drinking, residential radon exposure, past history of lung diseases, any cancer in first-degree relatives, intakes of meat, exposure to known or suspected lung carcinogens, and adoption of dust control

Supplementary Table 4. Studies included in the meta-analysis for passive smoking in women

Cancer site	Author (year)	Study period	Study subjects				OR (95% CI)	Confounding variables considered
			Type and source	Definition	No. of cases	No. of controls		
Lung	***Exposure to smoking at home, Incidence***							
	Jee SH et al. (1999) [40]	1992-1997	Korea Medical Insurance Corporation (KMIC)	Cohort<br>157,436 non-smoking wives<br><br>40 ≤ age ≤83	79		1.63 (1.01-2.63)	Adjusted for the age of husbands and wives, socioeconomic status, residency, husband's vegetable consumption, and husband's occupation.
	Kurahashi N et al. (2008) [42]	1990-1993	Japan Health Center-based Prospective study (JPHC Study)	Prospective cohort<br>28,414 non-smoking<br><br>40 ≤ age ≤69	109		1.24 (0.85-1.81)	Adjusted for age, study area, alcohol consumption, family history of lung cancer and menopausal status.
	Wen W et al. (2006) [56]	1997-2002	Shanghai Women's Health Study (SWHS)	Prospective cohort<br>72,829 non-smoking women	106		1.09 (0.74-1.61)	Adjusted for education, occupation, family income, physical activity, body mass index, and intake of meat, vegetables, and fruit.
	Nishino Y et al. (2001) [49]	1984-1992	Population-based	Prospective cohort<br>9,675 lifelong nonsmoking women	24		1.9 (0.81-4.4)	Adjusted for age
	Lee CH et al. (2000) [44]	1992-1998	Hospital-based	Cases: non-smoking female lung cancer patients<br>Controls: non-smoking women at the same hospital with conditions unrelated to tobacco use	268	445	2.2 (1.5-3.3)	Matched for age. Adjusted for residential area, education, occupation, tuberculosis, cooking fuels and fume extractor.
	Zhong L et al. (1999) [58]	1992-1994	Population-based Shanghai Residential Registry	Cases: non-smoker diagnosed with primary lung cancer<br>Controls: non-smoker selected randomly from the Shanghai Residential Registry	504	601	1.1 (0.8-1.5)	Adjusted for age, income, intake of vitamin C, respondent status, smokiness of the kitchen during cooking, family history of lung cancer, and potentially high-risk occupations.

Supplementary Table 4. Studies included in the meta-analysis for passive smoking in women (continued)

Cancer site	Author (year)	Study period	Study subjects				OR (95% CI)	Confounding variables considered
			Type and source	Definition	No. of cases	No. of controls		
Lung	***Exposure to smoking at home, Incidence***							
	Rapiti E et al. (1999) [50]	1991-1992	Hospital-based	Cases: non-smoker with newly diagnosed primary lung cancers Controls: non-smoker among patients admitted to the hospital and the patient's visitors	41	67	1.2 (0.5-2.9)	Adjusted for age, residence and religion.
	Wu-Williams AH et al. (1990) [57]	1985-1987	Cancer registries with community control	Cases: non-smoker with newly diagnosed primary lung cancers in cancer registries of Harbin and Shenyang Controls: non-smoker with randomly selected from the general populations of Harbin and Shenyang 50 ≤ age	417	602	0.79 (0.61-1.02)	
	Wang TJ et al. (1996) [55]	1992-1994	Hospital-based with community control	Cases: non-smoker with newly diagnosed of primary lung cancer in 18 hospitals of Shenyang Controls: non-smoker with randomly selected from the general population located in urban areas of Shenyang	135	135	1.11 (0.65-1.88)	Matched for age, sex
	Liu Q et al. (1993) [46]	1983-1984	Hospital-based	Cases: newly diagnosed cases of primary lung cancer from 8 hospitals Controls: individually matched hospital controls 40 ≤ age ≤70	38	69	1.49 (0.37-5.97)	Adjusted for education, occupation, and living area

Supplementary Table 4. Studies included in the meta-analysis for passive smoking in women (continued)

Cancer site	Author (year)	Study period	Study subjects				OR (95% CI)	Confounding variables considered
			Type and source	Definition	No. of cases	No. of controls		
Lung	***Exposure to smoking at home, Incidence***							
	Akiba S et al. (1986) [37]	1971-1980	Radiation Effects Research Foundation (RERF)	Cases: non-smoker with newly diagnosed cases of primary lung cancer among cohort members<br>Controls: non-smoker among cohort members without lung cancer	94	270	1.51 (0.85-2.77)	Matched for year of birth, city of residence, sex, and whether or not they were among the 20% of the cohort participating in the program of biennial medical examinations given at RERF, and vital status.
	Hirayama T (1984) [39]	1965-1981	Census-population-based	Prospective cohort<br>91,540 nonsmoking wives	200		1.59 (1.24-2.05)	Adjusted for age
	Lam TH et al. (1987) [43]	1983-1986	Hospital-based	Cases: non-smoking lung cancer patients<br>Controls: non-smoking healthy neighborhood controls matched for age	199	335	1.65 (1.16-2.35)	Matched for age
	Liu ZY et al. (1991) [47]		Population-based	Cases: newly-diagnosed lung cancer patients<br>Controls:	110 (total)	426 (total)	0.77 (0.30-1.96)	Matched for age, sex, occupation, and village of residence
	Fang J et al. (2006) [38]	2001-2004	Hospital-based	Cases: no-smoking female patients with the primary lung cancer from hospitals in Beijing, Shanghai and Chengdu<br>Controls: one hospital control and one population control			1.77 (1.07-2.92)	Matched for age, sex and no-smoking. Adjusted for consumption of internal organ, consumption of vegetables, taking vitamins often, average month income, and the first procreation age
	Wang L et al. (2000) [53]	1994-1998	Hospital-based with community control	Cases: diagnosed lung cancer among never-smokers<br>Controls: randomly selected from 1990 census lists	200	407	1.15 (0.6-2.1)	Frequency matched for age, sex, and prefecture of current residence

Supplementary Table 4. Studies included in the meta-analysis for passive smoking in women (continued)

Cancer site	Author (year)	Study period	Study subjects				OR (95% CI)	Confounding variables considered
			Type and source	Definition	No. of cases	No. of controls		
Lung	***Exposure to smoking at home, Incidence***							
	Shimizu H et al. (1988) [51]	1982-1985	Hospital-based	Cases: nonsmoking lung cancer patients<br>Controls: in-patients other than those with lung cancer<br>30 ≤ age	90	163	1.08 (0.64-1.82)	Matched hospital, age, date of admission
	Koo et al. (1987) [41]	1981-1983	Hospital-based with community control	Cases: never-smoked female lung cancer patients<br>Controls: never-smoked district controls	86	136	1.64 (0.87-3.09)	Adjusted for age, number of live births, schooling, years since exposure to cigarette smoke ceased in the home or workplace
	Exposure to smoking at home, Mortality							
	Lei YX et al. (1996) [45]	1986	Population-based	Cases: lung cancer deaths in non-smoker<br>Controls: non-tumor deaths in non-smoker	75	128	1.19 (0.66-2.16)	Matched for sex, age, year of death, and residence
	McGhee SM et al. (2005) [48]	1998	Death registries	Cases: deaths from lung cancer among never smoker<br>Controls: deaths among never smoking without lung cancer	179	345	1.38 (0.94-2.04)	Adjusted for age, education

.

Supplementary Table 4. Studies included in the meta-analysis for passive smoking in women (continued)

Cancer site	Author (year)	Study period	Study subjects				OR (95% CI)	Confounding variables considered
			Type and source	Definition	No. of cases	No. of controls		
Lung	***Exposure to smoking at workplace, Incidence***							
	Kurahashi N et al. (2008) [42]	1990-1993	Japan Health Center-based Prospective study (JPHC Study)	Prospective cohort 28,414 non-smoking	109		1.32 (0.85-2.04)	Adjusted for age, study area, alcohol consumption, family history of lung cancer and menopausal status.
	Wen W et al. (2006) [56]	1997-2002	Shanghai Women's Health Study (SWHS)	Prospective cohort 72,829 non-smoking women	106		1.79 (1.09-2.93)	Adjusted for education, occupation, family income, physical activity, body mass index, and intake of meat, vegetables, and fruit.
	Lee CH et al. (2000) [44]	1992-1998	Hospital-based	Cases: non-smoking female lung cancer patients<br>Controls: non-smoking women at the same hospital with conditions unrelated to tobacco use	268	445	1.2 (0.5-2.4)	Matched for age. Adjusted for residential area, education, occupation, tuberculosis, cooking fuels and fume extractor.
	Zhong L et al. (1999) [58]	1992-1994	Population-based Shanghai Residential Registry	Cases: non-smoking women diagnosed with primary lung cancer<br>Controls: non-smoking women selected randomly from the Shanghai Residential Registry	504	601	1.7 (1.3-2.3)	Adjusted for age, income, intake of vitamin C, respondent status, smokiness of the kitchen during cooking, family history of lung cancer, potentially high-risk occupations, and domestic exposure to environmental tobacco smoke.
	Wu-Williams AH et al. (1990) [57]	1985-1987	Cancer registries with community control	Cases: non-smoker with newly diagnosed primary lung cancers in cancer registries of Harbin and Shenyang<br>Controls: non-smoker with randomly selected from the general populations of Harbin and Shenyang	415	602	1.21 (0.94-1.58)	

Supplementary Table 4. Studies included in the meta-analysis for passive smoking in women (continued)

Cancer site	Author (year)	Study period	Study subjects				OR (95% CI)	Confounding variables considered
			Type and source	Definition	No. of cases	No. of controls		
Lung	***Exposure to smoking at workplace, Incidence***							
	Wang TJ et al. (1996) [55]	1992-1994	Hospital-based with community control	Cases: nonsmoker with newly diagnosed of primary lung cancer in 18 hospitals of Shenyang<br>Controls: non-smoker with randomly selected from the general population located in urban areas of Shenyang	135	135	0.89 (0.45-1.77)	Matched for age, sex
	Shimizu H et al. (1988) [51]	1982-1985	Hospital-based	Cases: nonsmoking lung cancer patients<br>Controls: in-patients other than those with lung cancer	90	163	1.20 (0.70-2.04)	Matched hospital, age, date of admission

Supplementary Table 5. Estimation of cancer incident cases and deaths attributable to passive smoking among non-smokers

		Lung cancer incidence		Lung cancer mortality	
		Men	Women	Men	Women
	Prevalence of tobacco smoking				
	% Current smokers	70.8	3.9	70.8	3.9
	% Former smokers	11.7	0.3	11.7	0.3
(A)	% Never-smokers	17.5	95.8	17.5	95.8
	AF estimate for secondhand smoking among never-smokers				
	Exposure to smoking at home				
	% Never-smokers exposed to smoking at home	14.8	60.1	14.8	60.1
	RR for lung cancer	1.00	1.32	1.34	1.32
(B)	PAF (%)	0.0	16.3	4.8	16.1
	Exposure to smoking at workplace				
	% Never-smokers exposed to smoking at workplace	42.2	14.7	42.2	14.7
	RR for lung cancer	1.15	1.37	1.15	1.37
(C)	PAF (%)	5.9	5.2	5.9	5.2
	Exposure to smoking at home or workplace				
(D)	PAF (%), (D)=(B)+(C)-(B)*(C)	5.9	20.7	10.5	20.5
	Number of cases attributable to secondhand smoking				
(E)	Total number of lung cancer cases in 2009	13,580	5,298	10,892	4,025
(F)	Lung cancer cases in smokers attributable to smoking	7,244	278	7,783	327
(G)	Lung cancer cases not attributable to smoking, (G)=(E)-(F)	6,336	5,020	3,109	3,698
(H)	Lung cancer cases among never-smokers, (H)=(G)*(A)/100	1,109	4,809	544	3,543
(I)	Lung cancer cases attributable to secondhand smoking at home, (I)=(H)*(B)/100	0	783	26	571
(J)	Lung cancer cases attributable to secondhand smoking at workplace, (J)=(H)*(C)/100	66	251	32	185
(K)	Lung cancer cases attributable to secondhand smoking at home or workplace, (K)=(H)*(D)/100	66	994	57	726
(L)	Total number of all cancer cases in 2009	96,826	91,068	43,658	25,773
	Exposure to smoking at home				
	% of lung cancer, (I)/(E) *100	0.0	14.8	0.2	14.2
	% of all cancers, (I)/(L) *100	0.0	0.9	0.1	2.2
	Exposure to smoking at workplace				
	% of lung cancer, (J)/(E) *100	0.5	4.7	0.3	4.6
	% of all cancers, (J)/(L) *100	0.1	0.3	0.1	0.7
	Exposure to smoking at home or workplace				
	% of lung cancer, (K)/(E) *100	0.5	18.8	0.5	18.0
	% of all cancers, (K)/(L) *100	0.1	1.1	0.1	2.8

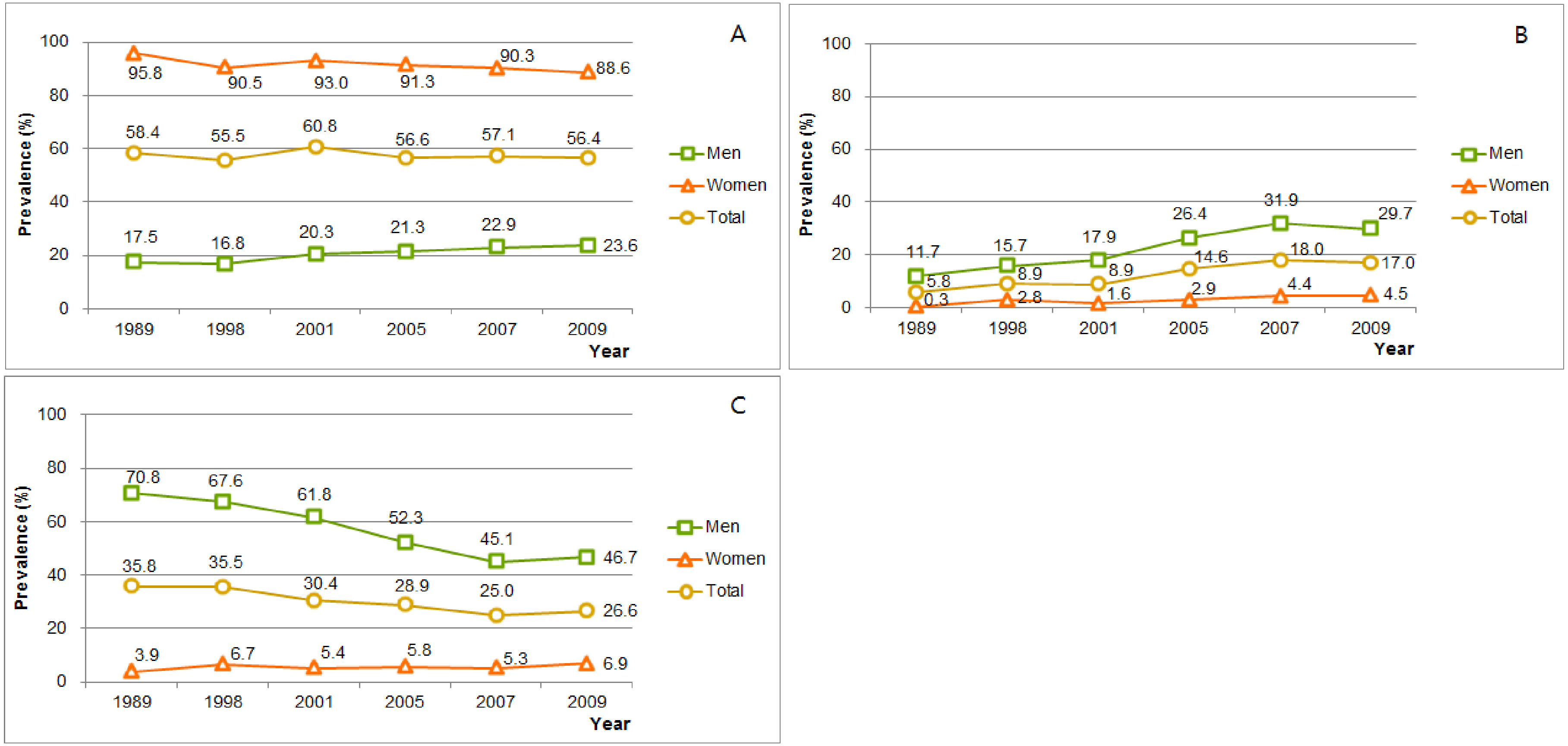

Supplementary Figure 1. Prevalence (%) of tobacco smoking. A) Never smoker, B) Former smoker, C) Current smoker

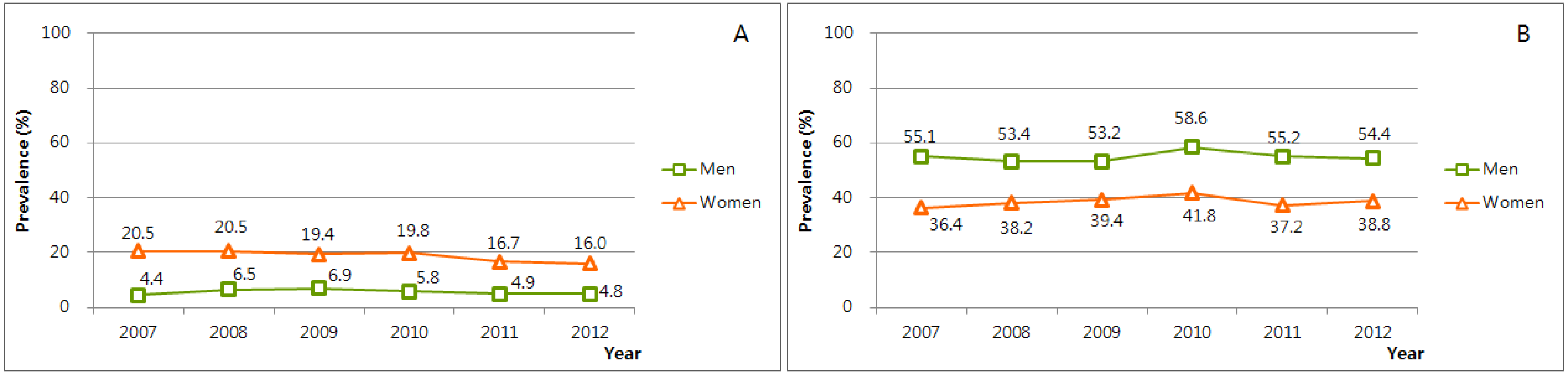

Supplementary Figure 2. Prevalence (%) of passive smoking A) At home, B) At workplace

Study name	Statistics for each study					Relative weight
	Odds ratio	Lower limit	Upper limit	Z-Value	p-Value	
Tse LA-2009	0.900	0.572	1.415	-0.456	0.648	76.78
Akiba S-1986	2.100	0.389	11.343	0.862	0.389	5.53
Wang L-2000	1.220	0.475	3.134	0.413	0.679	17.69
	0.995	0.669	1.480	-0.023	0.982	

Odds ratio and 95% CI

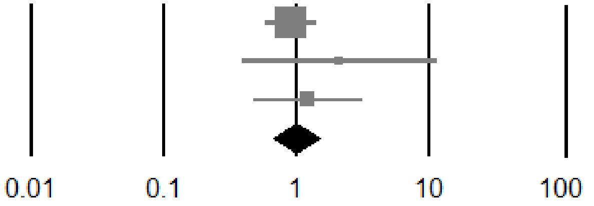

Footnote
Heterogeneity: χ^2 = 1.12 (d.f. = 2) p = 0.571; I^2 = 0.0%; Fixed effect estimate was selected.

Supplementary Figure 3. Meta-analysis on passive smoking at home and lung cancer incidence in men.

Study name	Odds ratio	Lower limit	Upper limit	Z-Value	p-Value	Relative weight
Jee SH-1999	1.630	1.010	2.630	2.002	0.045	5.81
Kurahashi N-2008	1.240	0.850	1.809	1.116	0.265	7.32
Wen W-2006	1.090	0.739	1.608	0.435	0.664	7.14
Nishino Y-2001	1.900	0.815	4.428	1.487	0.137	2.68
Lee CH-2000	2.200	1.483	3.263	3.921	0.000	7.05
Zhong L-1999	1.100	0.803	1.506	0.594	0.552	8.45
Rapiti E-1999	1.200	0.498	2.890	0.407	0.684	2.52
Wu-Wiliams-1990	0.790	0.611	1.021	-1.798	0.072	9.54
Wang TJ-1996	1.110	0.653	1.888	0.385	0.700	5.15
Liu Q-1993	1.490	0.371	5.985	0.562	0.574	1.14
Akiba S-1986	1.510	0.836	2.726	1.367	0.172	4.51
Hirayama T-1984	1.590	1.237	2.044	3.617	0.000	9.65
Lam TH-1987	1.650	1.159	2.349	2.781	0.005	7.75
Liu ZY-1991	0.770	0.301	1.968	-0.546	0.585	2.26
Fang J-2006	1.770	1.071	2.924	2.230	0.026	5.50
Wang L-2000	1.150	0.615	2.152	0.437	0.662	4.17
Shimizu H-1988	1.080	0.640	1.821	0.289	0.773	5.25
Koo LC-1987	1.640	0.870	3.091	1.530	0.126	4.11
	1.324	1.134	1.547	3.542	0.000	

Odds ratio and 95% CI

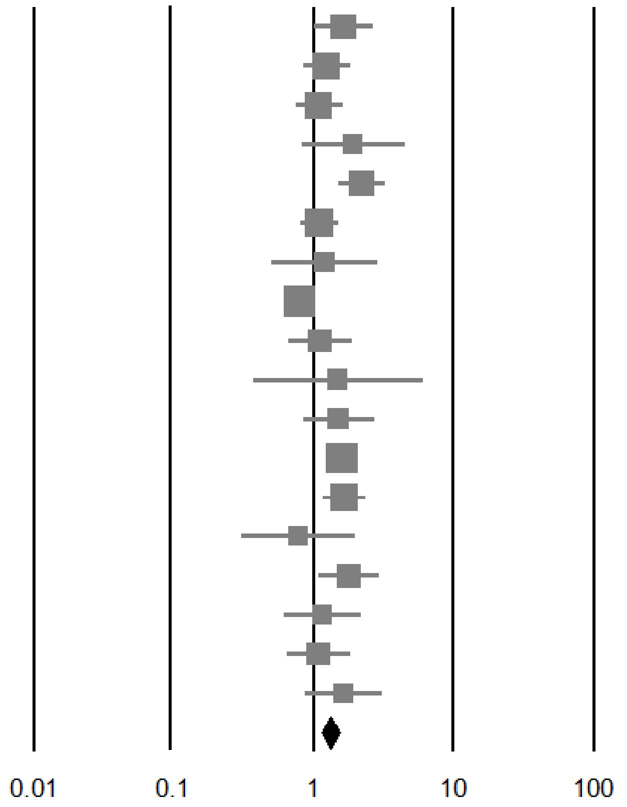

Footnote

Heterogeneity: $\chi^2 = 33.50$ (d.f. = 17) $p = 0.010$; $I^2 = 49.2\%$; Random effect estimate was selected.

Supplementary Figure 4. Meta-analysis on passive smoking at home and lung cancer incidence in women.

Study name	Odds ratio	Lower limit	Upper limit	Z-Value	p-Value	Relative weight
Lei YX-1996	1.190	0.658	2.153	0.575	0.565	29.93
Mcghee SM-2005	1.380	0.937	2.033	1.629	0.103	70.07
	1.320	0.954	1.826	1.678	0.093	

(Table headings: Study name; Statistics for each study; Odds ratio and 95% CI)

Odds ratio and 95% CI

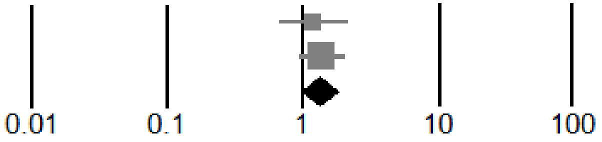

Footnote

Heterogeneity: $\chi^2 = 0.17$ (d.f. = 1) p = 0.682; $I^2 = 0.0\%$; Fixed effect estimate was selected.

Supplementary Figure 5. Meta-analysis on passive smoking at home and lung cancer mortality in women.

Study name	Odds ratio	Lower limit	Upper limit	Z-Value	p-Value	Relative weight
Kurahashi N-2008	1.320	0.852	2.045	1.243	0.214	11.95
Wen W-2006	1.790	1.092	2.935	2.308	0.021	9.36
Lee CH-2000	1.200	0.548	2.629	0.456	0.649	3.72
Zhong L-1999	1.700	1.278	2.261	3.646	0.000	28.13
Wu-Wiliams-1990	1.210	0.933	1.569	1.439	0.150	33.95
Wang TJ-1996	0.890	0.449	1.765	-0.334	0.739	4.88
Shimizu H-1988	1.200	0.703	2.049	0.668	0.504	8.00
	1.373	1.181	1.598	4.111	0.000	

(Column groups: **Study name**; **Statistics for each study**: Odds ratio, Lower limit, Upper limit, Z-Value, p-Value)

Odds ratio and 95% CI

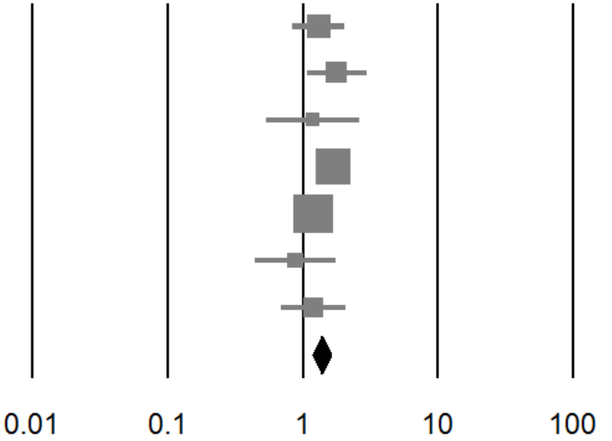

Footnote
Heterogeneity: χ^2 = 6.10 (d.f. = 6) p = 0.412; I^2 = 1.6 %; Fixed effect estimate was selected.

Supplementary Figure 6. Meta-analysis on passive smoking at workplace and lung cancer incidence in women.
